# Representing and querying disease networks using graph databases

**DOI:** 10.1186/s13040-016-0102-8

**Published:** 2016-07-25

**Authors:** Artem Lysenko, Irina A. Roznovăţ, Mansoor Saqi, Alexander Mazein, Christopher J Rawlings, Charles Auffray

**Affiliations:** 1 Rothamsted Research, Harpenden, West Common, Hertfordshire, AL5 2JQ UK; 2 European Institute for Systems Biology and Medicine (EISBM), CIRI UMR CNRS 5308, CNRS-ENS-UCBL-INSERM, Lyon, France

**Keywords:** Disease management platform, Graph database, Neo4j graph, Protein-centric framework, Systems medicine, Computational approach

## Abstract

**Background:**

Systems biology experiments generate large volumes of data of multiple modalities and this information presents a challenge for integration due to a mix of complexity together with rich semantics. Here, we describe how graph databases provide a powerful framework for storage, querying and envisioning of biological data.

**Results:**

We show how graph databases are well suited for the representation of biological information, which is typically highly connected, semi-structured and unpredictable. We outline an application case that uses the Neo4j graph database for building and querying a prototype network to provide biological context to asthma related genes.

**Conclusions:**

Our study suggests that graph databases provide a flexible solution for the integration of multiple types of biological data and facilitate exploratory data mining to support hypothesis generation.

**Electronic supplementary material:**

The online version of this article (doi:10.1186/s13040-016-0102-8) contains supplementary material, which is available to authorized users.

## Introduction

A major effort in translational medicine is to understand the molecular basis of disease [1, 2]. Analysis of high-throughput experimental data together with patient phenotypic information has led to the identification of sets of candidate genes, proteins and pathways that may be implicated in many disease conditions.

However, in order to build a higher level picture of the underlying processes involved in the disease pathology, it is necessary to integrate various classes of heterogeneous information, and to explore the complex relationships between entities such as diseases, candidate genes, proteins, interactions and pathways. These relationships could have a variety of different types, like *participation* (protein A participates in Pathway X), *sequence similarity* (protein A is sequence similar to protein B) or *protein interaction* (protein A interacts with protein B), and typically, will be associated with some provenance information. Generally, the relationships will describe complex networks. Some genes or proteins, for example, may be associated with multiple diseases, while others may be implicated in only one or two conditions. Additionally, some candidate genes may encode large families of sequence-similar proteins, and may thus be involved in multiple protein-protein interactions. Identification of these wider relationships between entities can help in providing biological context and in hypothesis generation on disease mechanisms. Traversing paths through the generated network can suggest hitherto unexpected relationships between entities, (e.g. relationships between disease conditions).

Biological information is typically highly connected, semi-structured and unpredictable, and these features are important in deciding an appropriate way to represent and query disease-relevant biomedical networks. Representing multiple types of relationships will generally lead to highly connected networks. Additionally, the amount of information associated with a given entity will not be uniform. Some proteins have been extensively investigated experimentally, are well annotated and associated with multiple complementary pieces of information. For example, a gene may be involved in a protein-protein interaction with another protein, may be a drug target for a particular drug, may be known to be enhanced in a particular tissue type or may participate in a particular pathway. Other proteins can have little or no information associated with them and are annotated only as ‘hypothetical proteins’. Therefore, such datasets do not naturally fit into a classic relational database model which would have been originally designed to handle application cases with a large number of uniform entries associated with a limited number of data types and representing relatively few relationships in each data model. Although highly connected and sparse networks can be stored in a relational database, generally traversal-type queries (joins), which connect data linked by different relationships, become too computationally expensive and cumbersome to design. Traversal type queries are important for hypothesis generation, as they can reveal paths connecting entities that might not have been expected, and might not be apparent from visual inspection of the network. Another issue is that biological data are often semi-structured, i.e. lacking a structured data model, while still possessing some semantic annotation. As relational databases require all data to be transformed to conform to a pre-defined schema, developing parsers for such semi-structured data can be particularly problematic. Biological research is also unpredictable in that new types of information can emerge with little advance notice – for example when new analytical instruments or new data resources are released. In a relational database management system, capturing these new data types often means re-design of a database schema, which is generally a complex and costly process. However, in a research setting this information is often of great importance and can impact on our understanding of the roles of genes and pathways in disease networks. A framework that facilitates the rapid and seamless inclusion of new data types would therefore be particularly well-suited to support the dynamic and ever-changing requirements of biomedical research community. Graph databases are a natural way to represent highly connected, non-uniformly distributed, semi-structured and unpredictable data as found in many biological systems studies [3, 4]. They also offer agile and flexible solutions and easily allow the inclusion of new data types.

Recently, there has been much interest in network representations of biological systems, particularly for network visualisation and network analytics. Several successful frameworks have been established and used to support bioinformatics analysis including, but not limited to, Cytoscape [5], Gephi [6] and NetworkX [7]. However, the tools currently available are primarily for visualisation and analysis of network properties and do not offer an in-built support for semantics, graph querying or the ability to effectively work with datasets that are too large to visualise. Graph databases can naturally complement such tools and methods, by offering this much-needed extra functionality.

Several graph-based approaches have been developed for biological data integration such as Biozon [8], BN++ [9], Ondex [10, 11], and BioMine [12]). Some approaches, although providing network views, use an underlying relational database (e.g. BN++). The Ondex platform is a data warehouse using a graph data model and has been applied to plant systems biology, a domain where the data is typically fragmented across a large number of data sources and often poorly annotated. More recently, Bio4j [4] uses a graph database (Neo4j) to integrate data from several major repositories such as Uniprot KB [13], Gene Ontology (GO) [14], RefSeq [15], NCBI Taxonomy [16], and Expasy Enzyme DB [17].

The need to move towards considering graphs as biological knowledge repositories is particularly evident in the biomedical domain. Many important diseases have now been extensively researched for decades and the amounts of knowledge accumulated are both vast and heterogeneous. The large volume of interconnected data means that its exploration using a network visualizer cannot be carried out easily and the heterogeneity of the data can make cost-effective management difficult in a relational database system.

In this work, we have explored the potential of using a graph database to facilitate data management and analysis in order to provide biological context to disease-related genes and proteins. This application case used the well-established Neo4j graph database for building and querying a prototype disease map for a complex disease, such as asthma. Neo4j has a free community edition that can be run as a lightweight desktop database as well as a database server, and an SQL-like versatile query language for graphs called Cypher. The database comes with an immediately available easy-to-use web interface that can display the results as both graphs and tables and also has interfaces to R, Python and Java - core languages of modern bioinformatics research.

We have integrated information from human protein-protein interactions, pathways, sequence similarity, disease-gene, gene-tissue and protein-drug associations. The selected examples are intended to illustrate how the Neo4j Cypher query language can be used to perform complex queries on this integrated dataset. We also show how information from gene expression studies can be easily added to the database and how the database can provide biological context for these differentially expressed genes. The main advantage of presented approach is in its ability to contextualise disease-associated genes by facilitating the identification and visual exploration of their network neighbourhood. In addition, as we have already explained, graph databases excel in traversal-type queries, facilitating the exploration of chains of connected concepts. This ‘link discovery’ can lead to new hitherto unexpected relationships being identified. Therefore, in our application case we have aimed to explore both of these data mining strategies, namely discovering connections between different entities of biomedical interest (queries in Listings 1, 2 and 5) and characterising the relevant context of diseases and biomarkers (queries in Listings 3 and 4).

The knowledgebase we constructed is organised according to the following principles. First, we integrated several typical multi-*omics* data (sequence similarity, transcriptomics metabolic pathways and protein-protein interactions), which provide a low-level perspective on the relationships between biological entities (genes, proteins and drugs). Then we have added the more general information that relates these lower-level entities to the higher-level processes – e.g. different diseases. The queries presented here are intended to provide illustrative examples about how to leverage connections across these two levels – for example, by discovering links in the multi-omics layer relevant to understanding the differences between different related diseases or using that information for suggesting drug targets.

## Methods

A graph database offers several options for representation of data. Connections between entities (i.e. nodes) can be encoded directly as edges linking them together (e.g. protein A interacts with protein B, where the edge between the nodes represents the ‘interacts with’ relationship). Additionally, properties can be associated with nodes (e.g. protein A has annotation X, where ‘has annotation X’ is a property of the node representing protein A), and nodes that share a given property could be understood to be linked. And lastly, nodes can be linked via edges to an intermediate node indicating a common property (e.g. membership in the same pathway, for example node i representing protein A is connected to node j, representing pathway P by an edge that describes the relationship ‘participates in’). For this application case, the choice was guided primarily by the concerns of efficiency in capturing the structure of the data. For example, a property is an appropriate choice when annotation is simple, like a category label. An edge can have properties of its own, so can capture situations where the relationship is qualified, like a protein-protein interaction with given methods (e.g. 2-hybrid, anti tag co-immunoprecipitation) and associated with a confidence score.

### Data sources imported

All data used in this application case were imported into the Neo4j v2.3.1 graph database. The integration was done from a protein-centric perspective, where the nodes corresponding to the individual entries in the human subset of UniProt/Swissprot served as key anchoring points for all other types of annotation. As many of the databases we used are continuously updated, the point of reference when all data were acquired for this study is 27/03/2015. Sequence similarity was determined by running all versus all Tera-BlastP^TM^ for the UniProt Human reference proteome on the Timelogic DeCypher system. The significance threshold was 1e − 05, with effective database size adjusted to 16.9 bln bases. The results were further processed by retaining bi-directional hits only and modelled in the graph database as sequence similarity edges. The set of sequence similarity data was refined to exclude duplicates based on the UniProt id pair: the similarity between protein1-protein2 and protein2-protein1 was represented by a single, undirected edge. A score was assigned to each edge, calculated as a negative log10 of the average of the two e-values; only hits for which the ratio between the alignment length and the shortest sequence was above 0.60 were included.

Protein-protein interaction data were imported from the EBI IntAct database [18] (PSI-MI tab format). Each interaction edge was annotated with a list of experimental methods supporting it, as well as a confidence score (as given by the IntAct database). Data about human metabolic pathways were acquired from the Reactome database (see for example [19]). Individual pathways were modelled as nodes, with edges linking them to proteins to indicate pathway membership. Data about protein association with particular diseases were modelled in a similar way. The diseases, as defined by the curated subset of DisGeNET [20] (2014 release), were likewise represented as nodes with edges connecting them to corresponding proteins; (mapping gene names to UniProt identifiers was done via the Restful web services API of the UniProt database). Data on approved drug compounds were downloaded from DrugBank [21] on 27/03/2015 as DrugBank csv files. Where such information was available, those compounds were linked to proteins they target via UniProt identifier references. Data on gene expression-tissue enhanced association have been downloaded from the Human Protein Atlas (HPA), (version 13), [22], and integrated in the database. The tissue types were represented by nodes in the graph database and the enhanced expression in particular tissue types were modelled as edges, with RNA-TS-FPKM values as property. Information on ENSEMBL ids, (used for gene identification within HPA), was mapped to UniProt ids using the ‘Retrieve/ID mapping’ tool from the UniProt database, and noted as a property of the Protein node.

We have included a set of high-confidence manually curated genes believed to be implicated in one of the respiratory diseases as identified by Kaneko et al. [23]. The review categorized genes into four partially overlapping subsets of Bronchial Asthma (BA), Chronic Obstructive Pulmonary Disease (COPD), Essential hypertension (E-HTN) and Tuberculosis (TB). TB and E-HTN are included in the list as controls [23]. The gene names given in the paper were manually resolved to the UniProt identifiers and imported as a list attribute of corresponding annotations. This resulted in 219 genes (104 BA, 58 COPD, 35 TB, 54 E-HTN); detailed information on these gene sets is given in Additional file 1: Table S4. This type of information, gathered typically from domain experts or from careful study of the literature, is important in disease network construction as it serves as a starting point to explore pathways and processes that may be associated with the disease phenotype.

**Fig. 1:**
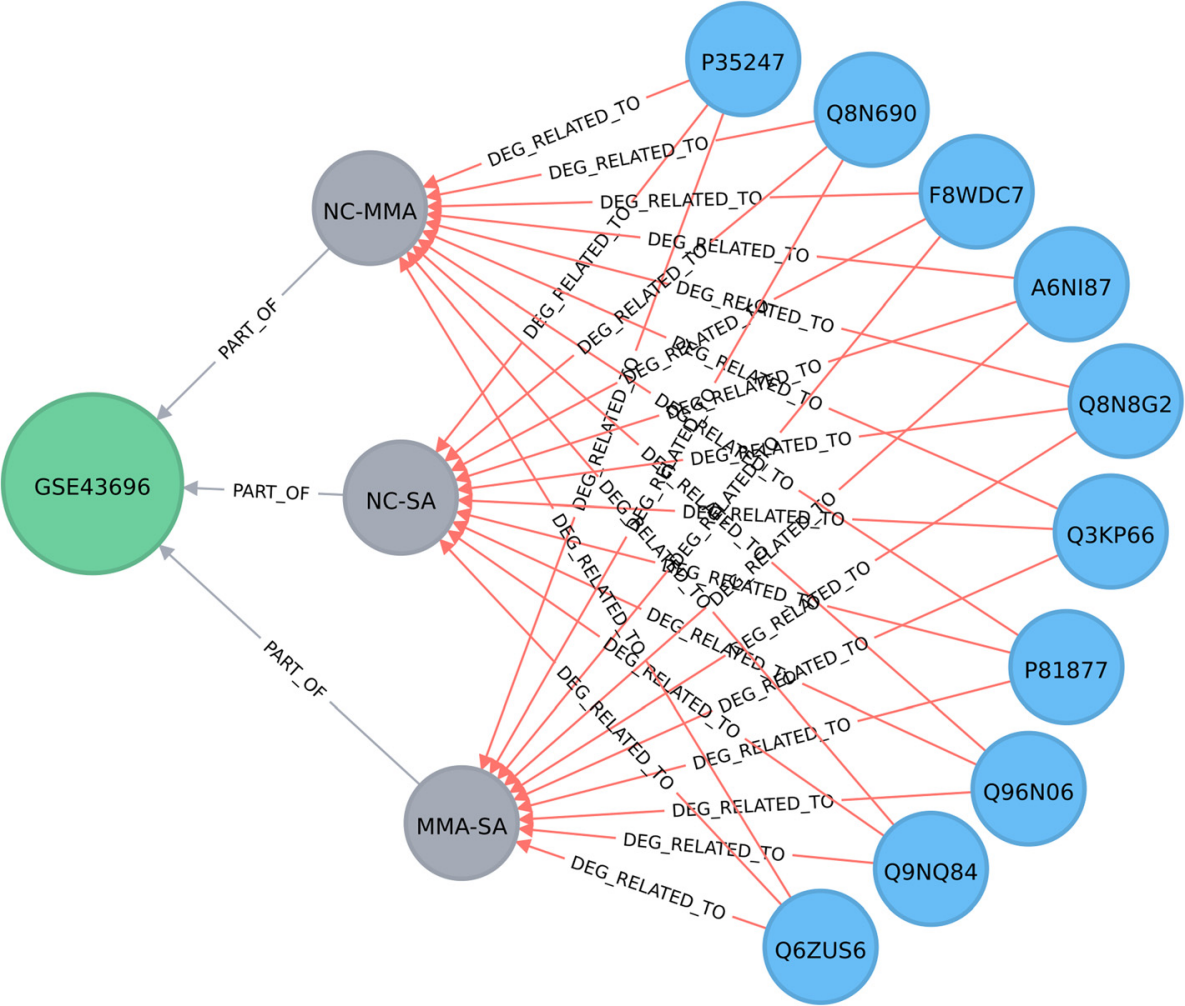
The data model for inclusion of results from gene expression studies. The Protein nodes, (*blue*), are associated to the GEO Comparison nodes (*grey*) by the DEG RELATED TO edges, (*red*); relationships between GEO Comparison and GEO Study nodes (*green*) are represented by the PART OF edges, (*green*). The key for i) the Protein node is given by the UniProt identifier, ii) the GEO Study node by its name and iii) the GEO Comparison node by the GSM samples that are compared. Information on adjusted p-values of the differentially expressed genes is stored as a property for the DEG RELATED TO edges. This simplified illustration includes only 10 differentially expressed proteins for the GSE43696 study [24], with similar representation for other studies. NC: normal control; MMA: mild-moderate asthma; SA: severe asthma

New experimental data may have significant impact on hypothesis generation on disease mechanisms. High-throughput transcriptomic experiments are a common original source of such new information, and are routinely used to identify lists of new potential candidate genes of biomedical interest. To evaluate the suitability of graph databases for management and integrative analysis of these data types we have included several such studies from the GEO database. We have extracted gene expression data from three asthma-related GEO studies, namely GSE27876, GSE43696 [24] and GSE63142 [25]. Specifically, the GSE27876 series includes 5 normal control (NC), 5 mild asthma (MiA) and 5 severe asthma (SA) samples, the GSE43696 series: 20 NC, 50 mild-moderate asthma (MMA) and 38 SA samples, and the GSE63142 series: 27 NC, 72 MMA and 56 SA samples. Gene expression data analysis was conducted on the GPL6480 platform [Agilent-014850 Whole Human Genome Microarray 4x44K G4112F (Probe Name version)], within all these series. The identification of the differentially expressed genes (DEGs) between asthma subtype/control cohorts was carried out using the *limma* Bioconductor package [26]. The gene name (given by ENSEMBL identifier) was mapped to the UniProt identifier, (again, via the ‘Retrieve/ID mapping’ tool from the UniProt database), with the UniProt identifier represented as a node in the graph database and the related ENSEMBL information as node property. Figure 1 illustrates how a node represents a GEO Study (e.g. GSE43696), which is connected to the 3 comparisons that are ‘part of’ the study. A ‘GEO Comparison’ node is related to the GSM samples (from the GSE series) and describes which sample groups are compared (e.g. normal control and mild-moderate asthma, NC - MMA). The Protein node is linked to the GEO Comparison node by the ‘DEG RELATED TO’ edge, (with the adjusted *p*-value of the DEG analysis). In this paper, the UniProt identifier gives the label of the Protein node, but other information (e.g. gene name) can be chosen from the Neo4j interface to be displayed as the label as well. Information on the ENTREZ gene name and the protein name, specific to the Protein nodes shown in Fig. 1, is given in Additional file 2: Table S5a.

**Fig. 2:**
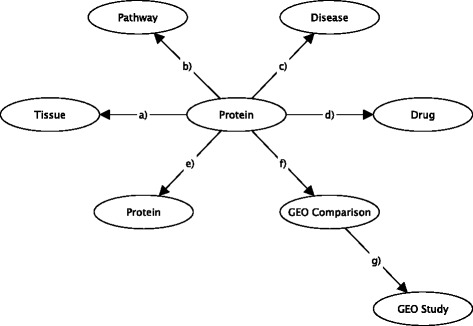
The Data Model: Schematic representation of biological information on proteins, pathways, tissues, disease and drugs, and the names of the GEO data sources, (GEO Study id, GSM sample type comparison), represented by nodes in the graph database. Relationships between these entities are shown by edges and refer to associations between: a) protein-tissue, (TISSUEENHANCED); b) protein-pathway (IN PATHWAY); c) protein-disease (BIOMARKER, GENETIC VARIATION, THERAPEUTIC, KANEKO ASSOCIATION); d) protein-drug (DRUG TARGET, DRUG ENZYME, DRUG CARRIER, DRUG TRANSPORTER), e) protein-protein (PPI ASSOCIATION, PPI COLOCALIZATION, PPI GENETIC INTERACTION, SEQ SIM); f) protein-GEO Comparison (DEG RELATED TO) and g) GEO Comparison-GEO Study (PART OF)

Figure 2 illustrates the structure of the graph database and Table 1 gives details of the data imported into the database in terms of nodes and edge types. Information to add was checked with already stored data before each adding step in order to avoid duplicates. Thus, the UniProt identifier, (i.e. the key of the Protein node), can be uniquely retrieved in the graph database. Similarly, names of diseases, pathways, tissues, GEO studies, (i.e. the keys for the Disease, Pathway, Tissue, GEO Study nodes, respectively), were checked for duplicates. The microarray data in the figure is also resolved to protein ids, so this information will be captured if available. E.g. some probe sets will profile alternative splice variants and will therefore map to different protein ids. Relationships between entities, (i.e. edges), were also checked for duplicates based on keys given by pairs of the involved entities; for example, the key of the Biomarker relationship between the Protein and Disease nodes is given by the pair composed from the UniProt identifier and disease name. Only when modelling associations between DEG and GEO comparison/study, the key was taken as a triplet composed from the UniProt identifier, the GEO Comparison name and the GEO Study identifier, given that the GEO comparison names are not unique among all GEO studies integrated in this database. Detailed information on node and edge occurrences is given in Table 1.

**Table 1:** Overall occurrences and types of nodes and relationships included in the graph database

(a) Occurrences of node types			
Node type			Occurrences
Protein			20762
Disease			4745
Pathway			1288
Tissue			32
Drug			1602
GEO Comparison_test			9
GEO Study			3
(b) Types and occurrences of relationships			
Node type	Edge type	Node type	Occurrences
Protein	BIOMARKER	Disease	17216
Protein	THERAPEUTIC	Disease	1209
Protein	GENETIC_VARIATION	Disease	2612
Protein	KANEKO_ASSOCIATED	Disease	251
Protein	IN_PATHWAY	Pathway	26085
Protein	PPI_ASSOCIATION	Protein	66678
Protein	PPI_COLOCALIZATION	Protein	1162
Protein	PPI_GENETIC_INTERACTION	Protein	43
Protein	SEQ_SIM	Protein	92089
Protein	TISSUE_ENHANCED	Tissue	4434
Protein	DRUG_TARGET	Drug	6429
Protein	DRUG_ENZYME	Drug	3204
Protein	DRUG_TRANSPORTER	Drug	1529
Protein	DRUG_CARRIER	Drug	222
GEO Comparison_test	PART_OF	GEO Study	9
Protein	DEG_RELATED_TO	GEO Comparison_test	87849

### Data modelling strategy

Unlike the relational database, where tables and relationships are heavily influenced by technical constraints formalized as “normalization rules”, graph databases offer much more flexibility and are more closely guided by the needs of the intended application. For this application case we have chosen the entities that correspond to either logical or real entities of biomedical interest. From the multi-omics perspective, the two physical entities central to our application case are proteins and drugs. These can be members of multiple thematic sets – e.g. the proteins can be grouped by disease involvement, tissue and metabolic pathway and drugs can be linked to particular diseases. To allow us to explore the potentially complex inter-relationships between these sets, they are also represented as nodes and therefore can be referenced directly as part of the query pattern. The edges are used to indicate both i) the membership of an entity in a particular set (e.g. protein participates in a pathway) and ii) existence of relationships between individual entities (e.g. protein interacts with other protein).

The level of the detail was chosen in accordance with the need of our intended application case. For example, in theory it may be valid to model an interaction as a concept rather than an edge and then link it to specific tissues to indicate whether it can occur there. However, by doing so an extra node and two edges will be introduced into the graph, which will increase the computational complexity of querying the database and make query construction more challenging for the user. In this case, due to the applied nature of the work presented here, it was therefore more appropriate to opt for a more generalized representation. However, we recognize that often it may be necessary to develop detailed representations in order to capture provenance and in the case of broad-content rather than thematically focused resources. To demonstrate how such a scenario can be accommodated in Neo4j, we have modelled transcriptomics component of our dataset in a way that captures all information about its provenance (GEOStudy node), experimental design (GEOStudy to GEOComparison relationship) and the analysis used (GEOComparison node) to process the data.

## Results

Here we illustrate the use of a graph database for facilitating hypothesis generation on disease mechanisms. We describe four queries related to asthma, a complex respiratory disease whose symptoms include airway inflammation and remodelling. Asthma is associated with multiple phenotypes and co-morbidities and has similarities to other respiratory diseases such as COPD. A significant number of asthma patients do not respond to conventional corticosteroid therapy, suggesting a complex underlying aetiology of this disease at both physiological and molecular levels.

### Identification of common proteins between Asthma and COPD, TB and E-HTN

**Fig. 3:**
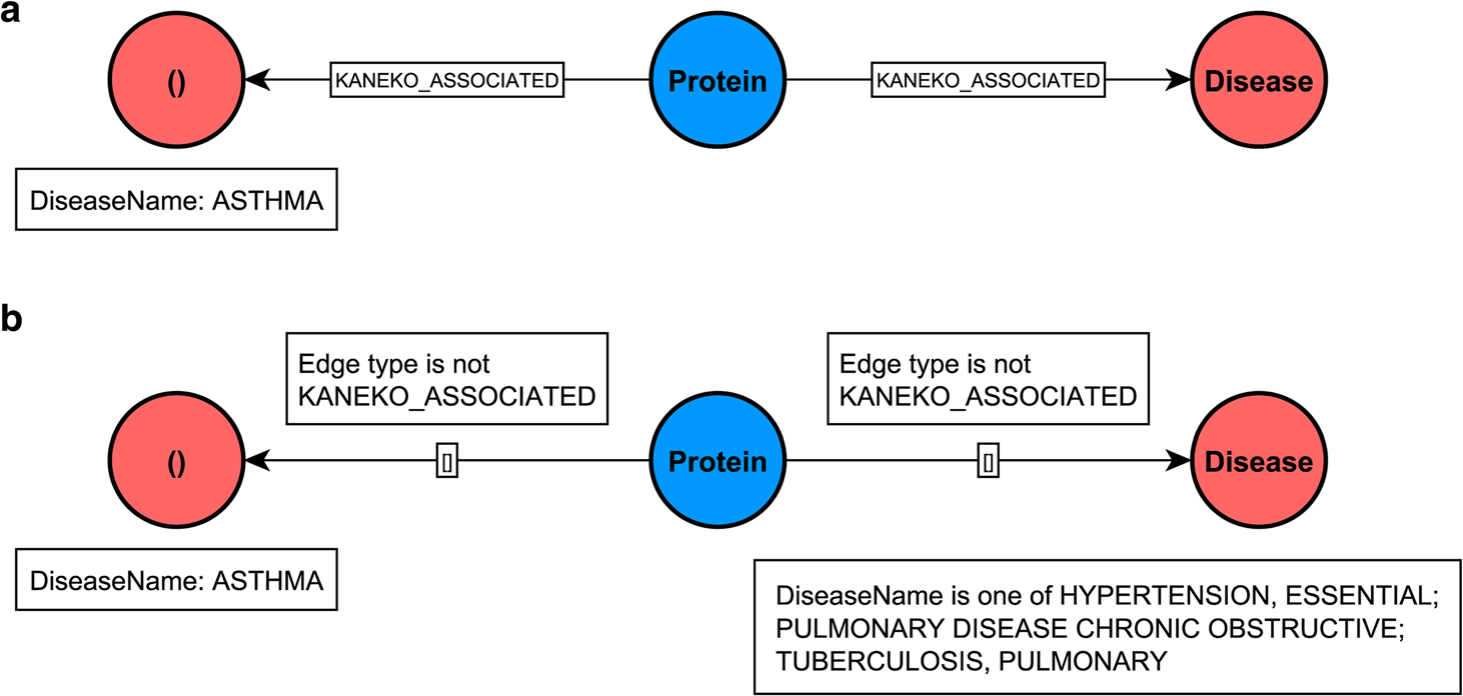
A visual schematic representation of graph patterns matched by queries in listing 1 (**a** panel) and 2 (**b** panel)

We first show a simple query that returns proteins, (given by UniProt identifiers), which are common to asthma and other respiratory diseases, (COPD, Tuberculosis (TB), and essential hypertension (E-HTN) respectively) using information from two sources, a review by Kaneko et al. (2013) [23] and, separately the DisGeNET database [20]. The Cypher queries are shown in Listings 1 and Listings 2, the visual schematic of the query pattern is shown on Fig. 3 and the results are shown in alphabetic order based on the second disease name in Table 2. Some details of the Cypher query are briefly explained in Additional file 3. We see more genes in common for asthma and COPD than for asthma and the other two respiratory diseases as expected, although differences are dependent on the data source used for comparison. While there is a degree of commonality among results from these resources, inclusion of multiple data sources can provide complementary information. Information on genes related to a simple disease only, as reported in [23], is provided in Additional file 4.

**Table 2:** Common protein set between Asthma and COPD, TB and EHTN, respectively, based on data from Kaneko et al. 2013 review [23] and the DisGeNET database [20]

Alter disease	Uniprot id set (Kaneko)	Uniprot id set (Disgenet)
Hypertension, Essential	[P29474, P04040, P12821, P07550, P30711]	[P35228]
Pulmonary Dis- Ease, Chronic Obstructive	[P01375, O00206, P01137, P35225, P01584, P29474, Q9BZ11, P07550, P12821, P09211, Q96QV1, P09488, P16410, P05305]	[P35228, P09601, P01137, P14780, P01375]
Tuberculosis, Pulmonary	[P01579, P01920, P01911, P01584, P22301, P29460, P05112, P13501, P11473, P01375, O60603, Q9NR96]	[P13500]

Listing 1: Cypher query to identify proteins common to asthma and other respiratory diseases using Kaneko et al. (2013) data [23]
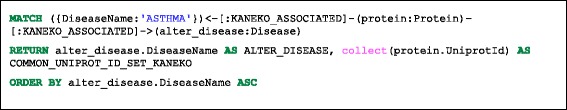


Listing 2: Cypher query to identify proteins common to asthma and other respiratory diseases using DisGeNET data only [18]
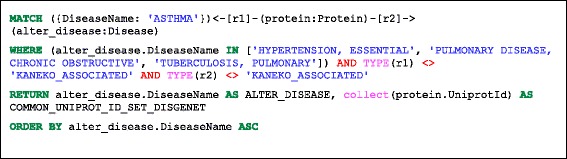


### Providing biological context: comparison of the network neighbourhoods of differentially expressed genes from two asthma studies

**Fig. 4:**
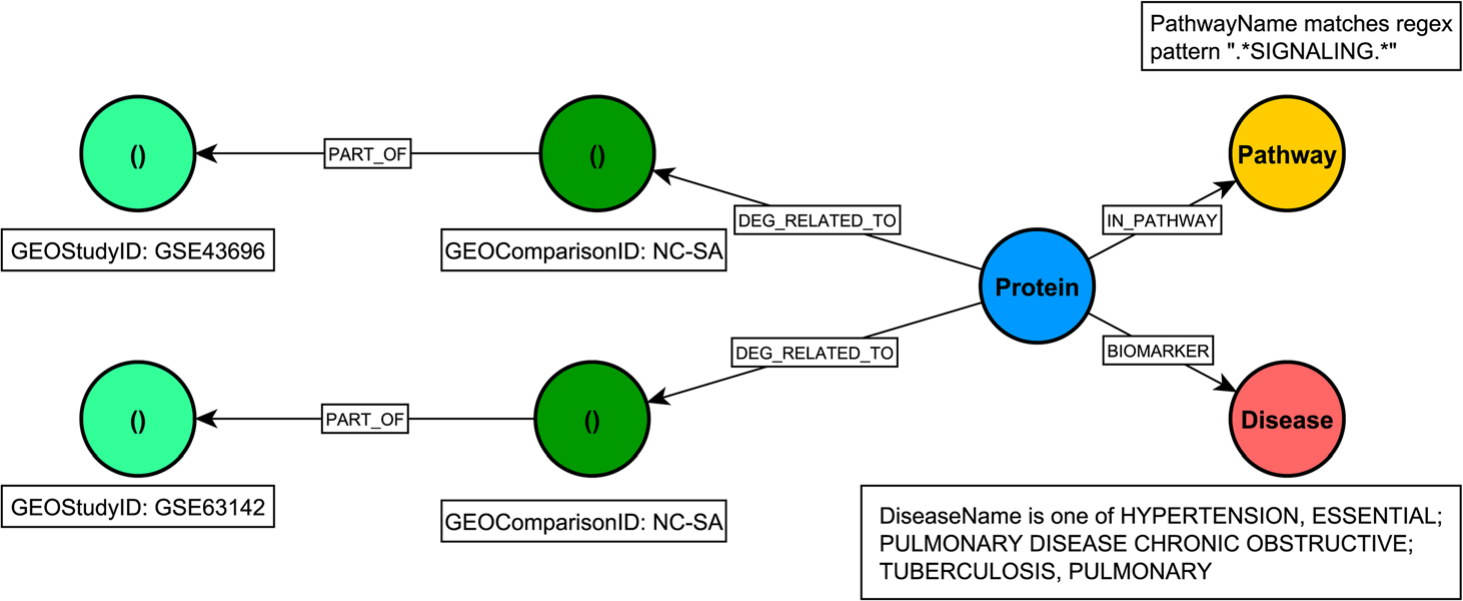
A visual schematic representation of graph pattern matched by query in listing 3

**Fig. 5:**
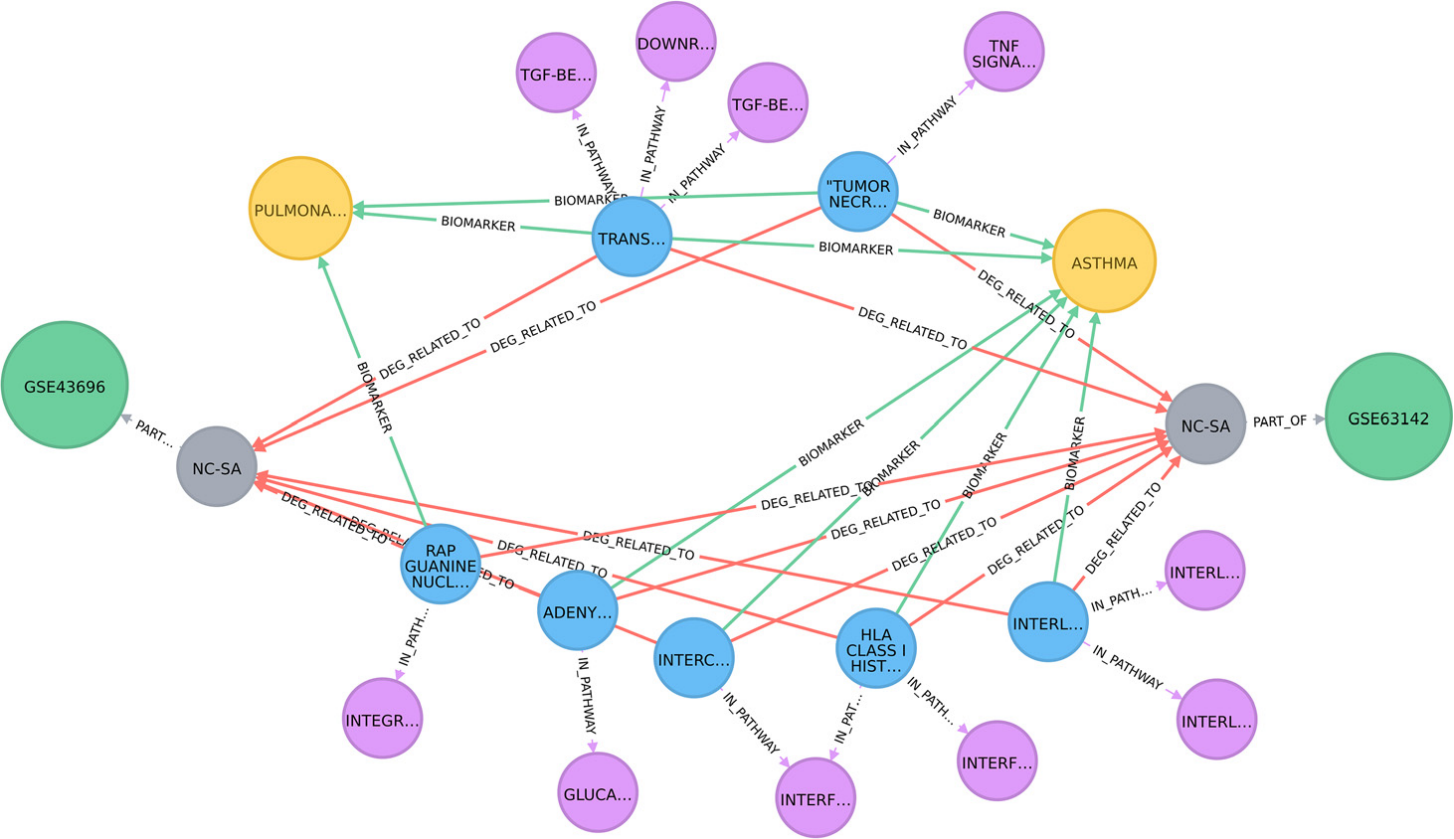
Disease – Protein-Signalling pathways associations: Common set of normal control - severe asthma DEGs for GSE43696 [24] and GSE63142 [25] series and their associations with respiratory diseases and signalling pathways. Node colour: protein, *blue*; GEO Comparison, *grey*; pathway, *violet*; disease, *yellow*. Edges: GEO comparison - GEO study relationship, *grey*; protein-pathway association, *violet*; DEG association, *red*; biomarker, *green*

Queries can easily be performed to select differentially expressed genes and relate them to the rest of the network. This example compares the network neighbourhoods of two sets of differentially expressed genes (DEGs) between Normal Control (NC) and Severe Asthma (SA) from two studies, GSE43696 [24] and GSE63142 [25]. In this example we have restricted the network neighbourhood to include only links from the DEGs common to these two studies, to Reactome signalling pathways [19] and to respiratory diseases. The Cypher query is shown in Listing 3, the corresponding pattern – in Fig. 4 and part of the resulting network is shown in Fig. 5. Similarly to Fig. 1, details on the ENTREZ gene name and the protein name of the Protein nodes (Fig. 5) are included in Additional file 2: Table S5b.

Listing 3: Cypher query to identify DEGs that map to asthma and other respiratory diseases as well as to signalling pathways.
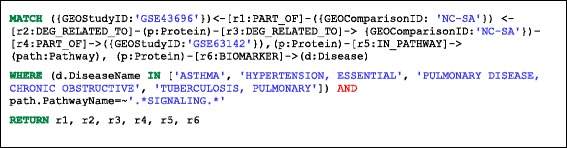


### Retrieval of drug associations with asthma-related proteins and other sequence similar proteins

**Fig. 6:**
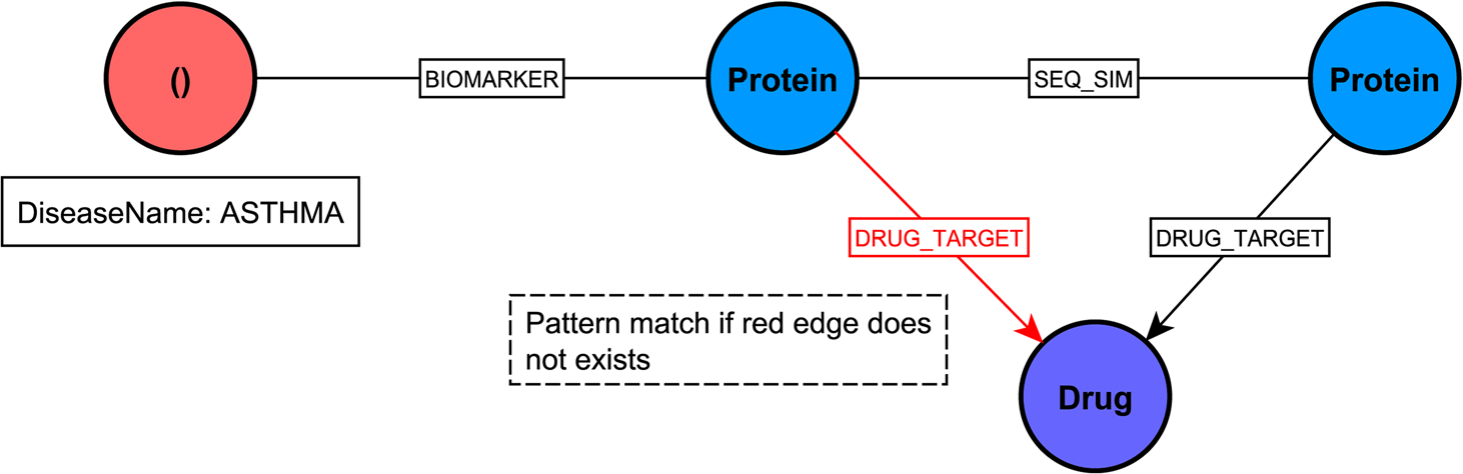
A visual schematic representation of graph pattern matched by query in listing 4

**Fig. 7:**
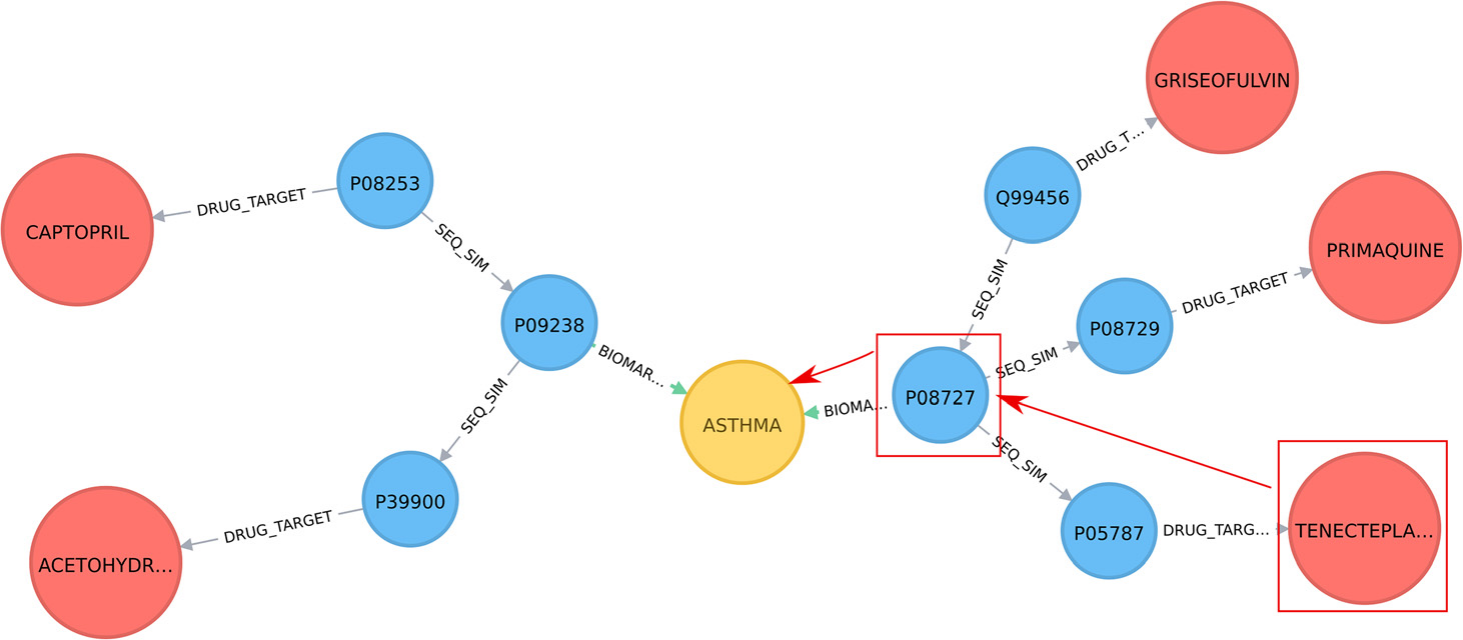
Drugs that target proteins, which have sequence similarity to asthma biomarkers. No information on direct target interaction between these drugs and biomarkers is given in the database a priori. Nodes colours: protein, *blue*; disease, *yellow*; drug, *red*. Edges: drug-target associations, *red*; sequence similarity relationships, *grey*; biomarker, *green*

We have queried for a set of drugs from DrugBank DB that target proteins of similar sequence to known asthma biomarkers (associated to asthma disease through the Biomarker relationship in DisGeNET). The query, pattern and part of the relevant network are shown in Listing 4, Figs. 6 and 7, respectively. The query can be easily extended to include other disease-protein relationship types, (e.g. Therapeutic), other disease types, (e.g. COPD), and filters for output, (e.g. for sequence similarity better than a threshold value).

Listing 4: Cypher query to identify drugs associated with proteins that have sequence similarity with asthma biomarkers.
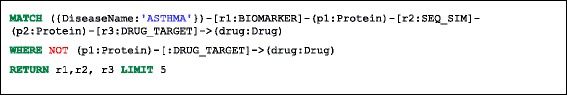


Results obtained can be used to infer new associations between drugs and asthma biomarkers. For example, although no direct association has been reported for the P08727 protein (asthma biomarker) and Tenecteplase (drug), an indirect relationship can be identified between these two entities given that the P05787 protein (*KRT8* gene) has sequence similarity with the P08727 protein, which was reported as a target for Tenecteplase, (Fig. 7). Exploring such indirect relationships can be of interest in drug development research. The ENTREZ gene name and the protein name for Fig. 7 are provided in Additional file 2: Table S5c.

### Graph traversal queries for exploring relationships between concepts

**Fig. 8:**
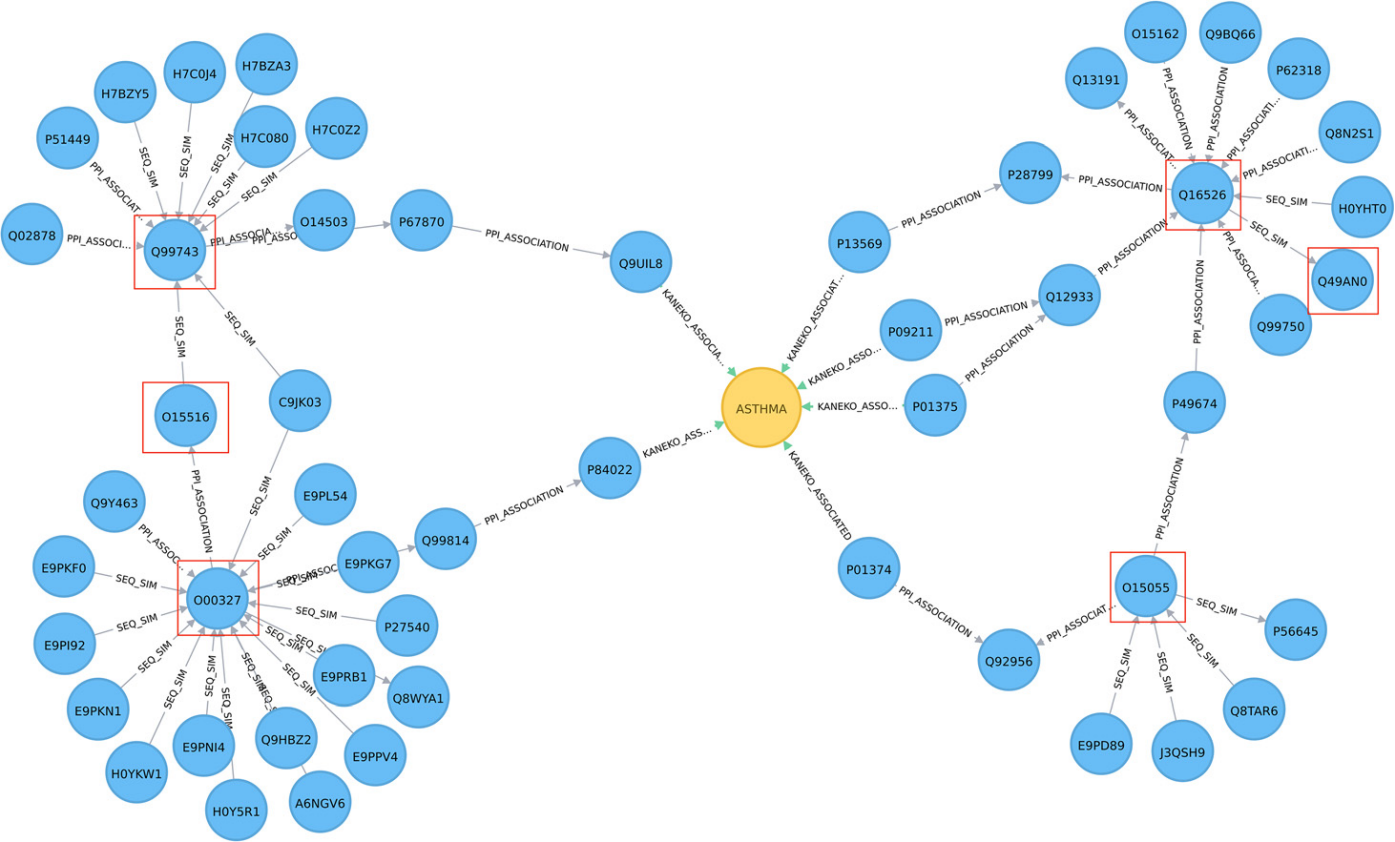
Shortest paths (of length < 4) between core clock components (*red squares*) and asthma-related proteins in the network. Node colour: disease, *yellow*; protein, *blue*. Edges: KANEKO association, *blue*; PPI association, *red*; sequence similarity relationship, *grey*

Studies suggest that asthma patients may experience exacerbations early in the morning (see for example [27]). Independently, considerable research efforts have been directed towards identification of genes involved in mediating circadian rhythms (’clock’ genes). Exploration of the relationships between circadian system disruption and lung disease development, (including asthma), may identify new potential targets (e.g. proteins, pathways) for disease treatment and may give better insights in drug administration [27, 28]. The following example identifies all shortest paths in the graph between asthma disease and a subset of core clock components (protein-coding genes that generate and regulate circadian rhythms), where the corresponding UniProt identifiers were resolved using the “Retrieve/ID mapping” UniProt tool (Table 3). Results are shown in Fig. 8, based on the Cypher query in Listings 5, with the ENTREZ gene names and the protein names given in Additional file 2: Table S5d. This example illustrates how simple graph traversal queries have the potential to assist in hypothesis generation by exploring relationships between concepts.

**Table 3:** Gene symbols and corresponding UniProt identifiers, (resolved via the “Retrieve/ID mapping” tool), for the core circadian components

Gene symbol	UniProt identifier
CLOCK	O15516
ARNTL/BMAL1	O00327
CRY1	Q16526
CRY2	Q49AN0
NPAS2	Q99743
PER1	O15534
PER2	O15055

Listing 5: Cypher query to explore shortest paths (in terms of graph representation) between core clock genes and asthma.
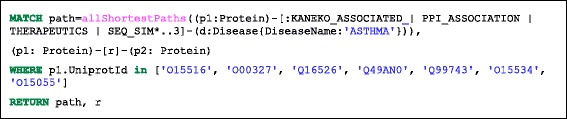


**Fig. 9:**
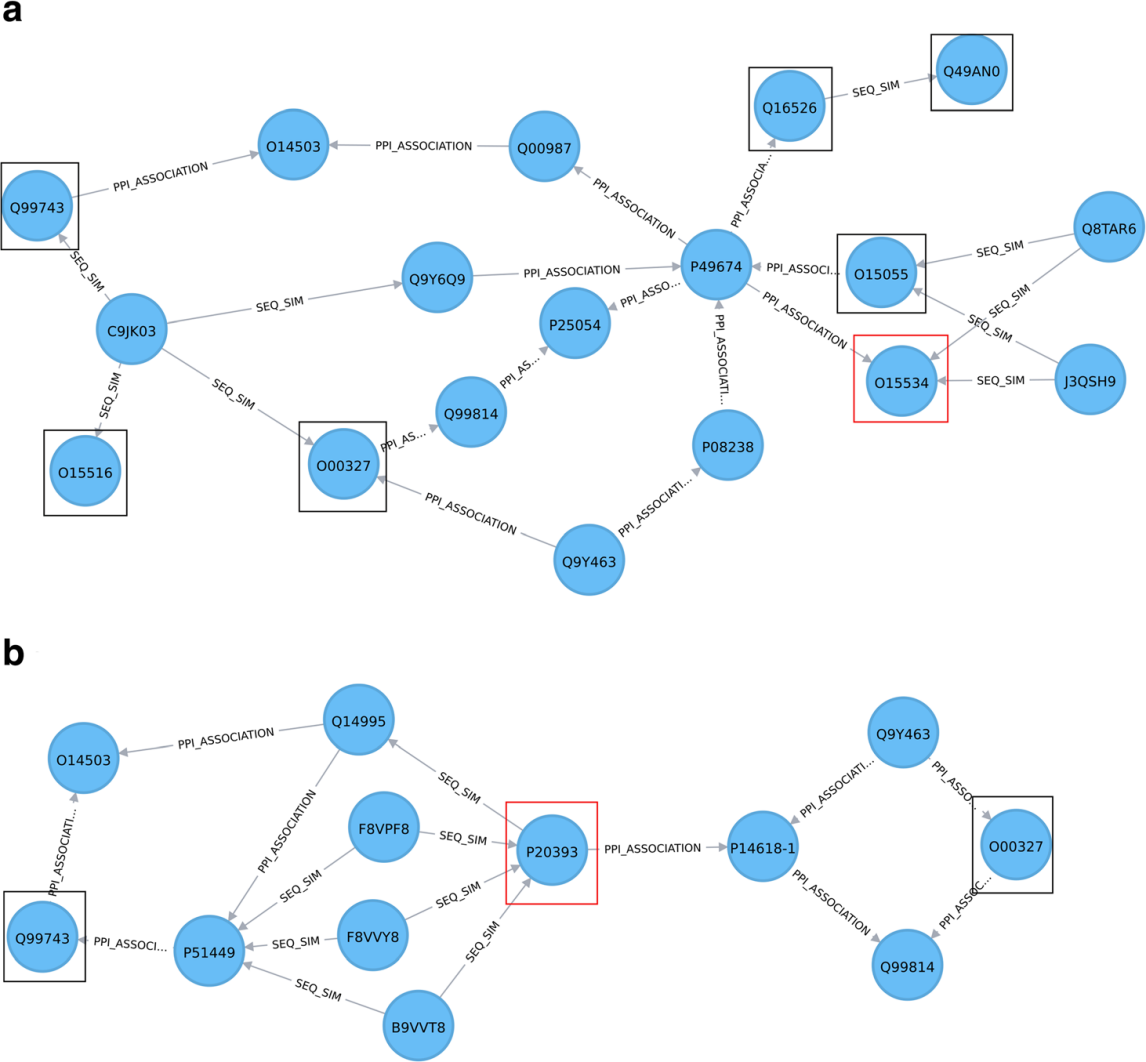
Shortest path queries to explore relationships between a) the O15534 protein (PER1 gene) and b) the P20393 protein (REV-ErbA-alpha gene) and the circadian core genes, (Table 3). **a** In terms of distance in graph, the O15534 protein (PER1 gene) (red square), transcriptional repressor, is closer to O15055 (PER2), Q16526 (CRY1) and Q49AN0 (CRY2), (transcriptional repressors), than to O15516 (CLOCK), O00327 (ARNTL/BMAL1) and Q99743 (NPAS2), (transcriptional activators). The circadian core genes are shown by *black squares*. **b** The P20393 protein (REV-ErbA-alpha gene) (*red square*), suggested to be involved in the disruption of clock genes [27], can be seen 3 steps away from the O00327(ARNTL/BMAL1) and Q99743(NPAS2) core clock genes (*black squares*). Node colour: protein, *blue*. Edges: PPI association, *red*; sequence similarity relationship, *grey*

Shortest pathways (Fig. 8) between the Asthma node and the core clock genes (Table 3) node include: a) Asthma – Q9UIL8 – P67870 – Q99743; b) Asthma – P84022 – Q99814 – O00327; c) Asthma – P01374 – Q92956 – O15055; d) Asthma – [P09211, P01375, P13569] – [Q12933, P28799] – Q16526. The O15516 protein (encoded by the *CLOCK gene*) is linked through Sequence Similarity and PPI relationships by Q99743 (*NPAS2*) and O00327 (*ARNTL*/*BMAL1*), respectively, and the protein Q49AN0 (*CRY2*) has sequence similarity with Q16526 (*CRY1*). Interestingly, the O15534 protein, encoded by *PER1*, also a core clock gene, (Table 3), does not lie within 3 steps of Asthma and is not an immediate neighbour of any of the other core clock components in Table 3, that are within 3 steps of Asthma, (Fig. 8). We further queried for the shortest paths between O15534 (*PER1*) and the rest of the core clock gene subset (Table 3), using only association types related to protein-protein relationships, such as sequence similarity and PPI; the Cypher query is given in Listings 6a, in Additional file 5. The resultant network indicates that, in terms of topological distance, *PER1* is closer to *PER2*, *CRY1* and *CRY2* than to *CLOCK*, *ARNTL*/*BMAL1* and *NPAS2* (refer to Fig. 9a).

Durrington et al. [27] suggest the possibility of the disruption of clock genes affecting airway inflammation through a possible link of clock proteins to the immune response involving *REV*-*ErbA*-*alpha*. The graph network can be queried for P20393 (*REV*-*ErbA*-*alpha*) which does not appear on the shortest paths network in Fig. 8, but lies 3 steps from O00327(*ARNTL*/*BMAL1*) and Q99743(*NPAS2*), (Cypher query given in Listings 6b, in Additional file 5 and output results in Fig. 9b).

## Discussion and conclusions

High volume datasets are being routinely generated in systems biomedicine. An on-going concern for the interpretation of these data is that biological information has a challenging mix of complexity and rich semantics. Modelling of data in graph database ideally are guided by the same principles as those used for ontology design. In a way, the ontology may play the same role as a schema plays in a relational database by defining a valid set of relationships and/or properties that can exist for particular entities or their combinations. However, for this application case we have elected not to explore this topic in great detail in order to keep focus firmly on the applied aspects of managing biomedical data using the Neo4j database and to present it at a level accessible to a more general audience. For completeness, we would like to point out that from the ontology-centric data modelling perspective a Neo4j graph can be thought of as a collection of instances of data, where node “Labels” are equivalent to classes, and types of edges – to relationship types. Another important consideration in choosing how to represent the data is the performance constraints. [Note: We would encourage developers to look at the Neo4j features relating to Cartesian product warning and to query flow diagnosis, using the EXPLAIN and PROFILE features provided.] In this respect, as we were specifically interested in exploring the applications of the link discovery type of queries in a biomedical setting, it was necessary to ensure both the compactness of paths and adequate annotation necessary to ensure that the traversal space can be computed, e.g. it does not produce an all-versus-all set of entities as part of computing the solution (a “Cartesian product”).

In traditional relational databases approaches, the technical structure often gets in the way of exploratory data analysis either by visualisation or through data mining techniques. Graph databases (explored here with Neo4j) offer a powerful but lightweight, intuitive and flexible solution that is more readily compatible with a biological network view, a representation that is now near-ubiquitously employed for modelling complex biological data. This study shows how a combination of a powerful query language (to narrow down the search space) with network visualisation can help data exploration and hypothesis generation. The use of graph database approaches has some similarities with previous systems for logic-based modelling of integrated datasets [29, 30].

As we have illustrated in this work, graph databases offer many features that make them a particularly attractive option for a research-based setting, in particular in cases where it might be necessary to dynamically develop and interactively mine heterogeneous and not uniformly inter-related data. However, we would like to stress that in our opinion graph databases are best thought of as a complement to relational database technologies. There will clearly be cases where a relational database solution will be preferable, especially when data are dense and naturally fit the tabular representation where relational databases would offer much better processing performance.

Another important consideration is the place of Neo4j in a wider family of NoSQL and especially graph databases. The concept of graph databases is still relatively novel and therefore currently there are multiple alternative systems under active development. However, one standard – Resource Description Framework (RDF) and its enabling technologies (triple stores and SPARQL query language) have gained particular prominence due to their importance for Semantic Web and Internet-powered federated data solutions. For this reason we would like to further elaborate on the differences between these two standards and specifically the roles they could potentially play in management of large biomedical datasets.

Neo4j database belongs to the family of “Property graph” databases, which in contrast to RDF have conceptually distinct property elements that can be attached to both nodes and edges. In some respects, this syntax may be more familiar to users of object-oriented languages, as in contrast to RDF, there is neither a global uniqueness constraint nor specifically required format for node identifiers. In combination, these features make graph query syntax more compact and easier to read. Given that the Neo4j database is inherently a “closed world” solution, it is possible to collect the entirety of meta-data about the types and number of different entities and relationships between them, which can then inform further exploration. As SPARQL and RDF are primarily designed to operate in an “open world” setting of the Semantic Web, creating such summaries may not always be possible or can be very computationally intensive.

However, we would like to acknowledge that the RDF formalism does have its undisputed advantages in facilitating integration and exchange of distributed data and issues like lack of properties on edges can be addressed by leveraging more advanced features like reification or named graphs. In our view, the matter of preferences between the properties databases like Neo4j or RDF triple stores comes down to whether a particular application case can actually benefit from the extra features available via Semantic Web technologies. As adopting such technologies will inevitably come with considerable costs in terms of technical overhead and increased complexity, property databases may be preferable when speedy and flexible development of a single non-federated resource is the ultimate requirement.

The lack of schema in Neo4j may also be somewhat of a double-edge sword: although it does offer much flexibility, it also removes the interoperability standard from the data, which may make sharing and management of large distributed projects datasets more challenging. One possible solution to this would be to re-introduce some consistent semantics by binding critical attribute names and/or values of nodes and edges to a consensus controlled vocabulary. In a biomedical domain, this controlled vocabulary will likely come in the form of a suitable ontology. The ontologies could then provide a de-facto data modelling standard and many key concepts in the biomedical domain have already been modelled by projects like SNOMED [31], MeSH [32], GO [33] and OBI [34], with several hundred of other more specialized ontologies available from the OBO foundry [35] and NCBO Bioportal [36] repositories. Although at present the availability of advanced ontology-based features, like reasoning and data validation, varies in different graph databases, some notable solutions do offer examples of very close integration - e.g. the already-mentioned graph databases based on the resource description framework (RDF) [37].

We have explored here the utility of a graph database in providing a powerful yet flexible solution for disease network representations and for exploratory data mining and analysis to support hypothesis generation on disease mechanisms. Our study suggests that the Neo4j system offers a level of performance and an appropriate query and visualisation interface to effectively mine and manipulate these data. In particular, we have found the Cypher graph query language to be of great utility, because, as illustrated by our sample queries, it enabled us to generate a representative selection of common biologically-motivated queries with minimal efforts. As systems medicine projects continue to generate large amounts of heterogeneous datasets, graph database approaches may offer useful solutions for their knowledge management.

## Abbreviations

BA, bronchial asthma; COPD, chronic obstructive pulmonary disease; DEG, differentially expressed genes; E-HTN, essential hypertension; GEO, Gene Expression Omnibus (database); GSM, samples from the GEO database; HPA, Human Protein Atlas; MiA, mild asthma; MMA, mild-moderate asthma; NC, normal control; RDF, the resource description framework; SA, severe asthma; TB, tuberculosis

The ENTREZ gene names and the protein names are given in Additional file 2.

## Additional files

Additional file 1: Disease-associated genes used in the study (from [23]). (DOCX 93 kb) Additional file 2: ENTREZ gene name and protein names specific to the Protein nodes indicated in Figs*.* 1, 5, 7, 8 and 9. (DOCX 106 kb) Additional file 3: Brief introduction to Cypher query language; for more details on the Cypher language, the reader is referred to the Neo4j website: http://neo4j.com/. (DOCX 17 kb) Additional file 4: Information on genes related to a simple disease only, as reported in [23]. (DOCX 120 kb) Additional file 5: Cypher query on the relationships between a) the O15534 protein (PER gene) and b) the P20393 protein (REV/ERBalpha gene) and the core clock genes, (Table 3). (DOCX 77 kb)
