# Supplementary material for: Representing and querying disease networks using graph databases
Source: BioData Min. 2016 Jul 25;9:23. doi: 10.1186/s13040-016-0102-8 (PMC4960687; doi:10.1186/s13040-016-0102-8)
Supplement: Additional file 1: — Disease-associated genes used in the study (from [23]). (DOCX 93 kb) [file 13040_2016_102_MOESM1_ESM.docx]

Additional file 1 — Disease-associated genes used in the study (from [23])

1. Disease-associated genes used in the study (from [23])
2. Intersection of BA and COPD

| **Uniprot ID** | **Entrez Gene Name from Kaneko** | **Protein Name from UNIPROT** |
| --- | --- | --- |
| Q9BZ11 | ADAM33 | Disintegrin and metalloproteinase domain-containing protein 33 |
| P16410 | CTLA4 | Cytotoxic T-lymphocyte protein 4 |
| P01584 | IL1B | Interleukin-1 beta |
| P29474 | NOS3 | Nitric oxide synthase, endothelial |
| P01137 | TGFB1 | Transforming growth factor beta-1 |
| P05305 | EDN1 | Endothelin-1 |
| Q96QV1 | HHIP | Hedgehog-interacting protein |
| P09488 | GSTM1 | Glutathione S-transferase Mu 1 |
| P35225 | IL13 | Interleukin-13 |
| P12821 | ACE | Angiotensin-converting enzyme |
| P01375 | TNFA | Tumor necrosis factor |
| P07550 | ADRB2 | Beta-2 adrenergic receptor |
| P09211 | GSTP1 | Glutathione S-transferase P |
| O00206 | TLR4 | Toll-like receptor 4 |

1. Intersection of BA and EHTN

| **Uniprot ID** | **Entrez Gene Name from Kaneko** | | **Protein Name from UNIPROT** |
| --- | --- | --- | --- |
| P04040 | | CAT | Catalase |
| NA | | MBL |  |
| P12821 | | ACE | Angiotensin-converting enzyme |
| P29474 | | NOS3 | Nitric oxide synthase, endothelial |
| P30711 | | GSTT1 | Glutathione S-transferase theta-1 |
| P07550 | | ADRB2 | Beta-2 adrenergic receptor |

1. Intersection of BA and TB

| **Uniprot ID** | **Entrez Gene Name from Kaneko** | | **Protein Name from UNIPROT** |
| --- | --- | --- | --- |
| P22301 | | IL10 | Interleukin-10 |
| P05112 | | IL4 | Interleukin-4 |
| P11473 | | VDR | Vitamin D3 receptor |
| P13501 | | CCL5 | C-C motif chemokine 5 |
| P01584 | | IL1B | Interleukin-1 beta |
| Q9NR96 | | TLR9 | Toll-like receptor 9 |
| P01579 | | IFNG | Interferon gamma |
| P29460 | | IL12B | Interleukin-12 subunit beta |
| P01920 | | HLA-DQB | HLA class II histocompatibility antigen, DQ beta 1 chain |
| P01375 | | TNFA | Tumor necrosis factor |
| O60603 | | TLR2 | Toll-like receptor 2 |
| P01911 | | HLA-DRB1 | HLA class II histocompatibility antigen, DRB1-15 beta chain |

1. Intersection of COPD and EHTN

| **Uniprot ID** | **Entrez Gene Name from Kaneko** | | **Protein Name from UNIPROT** |
| --- | --- | --- | --- |
| P07550 | | ADRB2 | Beta-2 adrenergic receptor |
| P09601 | | HMOX1 | Heme oxygenase 1 |
| P12821 | | ACE | Angiotensin-converting enzyme |
| P29474 | | NOS3 | Nitric oxide synthase, endothelial |

1. Intersection of COPD and TB

| **Uniprot ID** | **Entrez Gene Name from Kaneko** | | **Protein Name from UNIPROT** |
| --- | --- | --- | --- |
| P01584 | | IL1B | Interleukin-1 beta |
| P01375 | | TNFA | Tumor necrosis factor |

1. Intersection of BA, COPD and EHTN

| **Uniprot ID** | **Entrez Gene Name from Kaneko** | **Protein Name from UNIPROT** |
| --- | --- | --- |
| P07550 | ADRB2 | Beta-2 adrenergic receptor |
| P29474 | NOS3 | Nitric oxide synthase, endothelial |
| P12821 | ACE | Angiotensin-converting enzyme |

1. Intersection of BA, COPD and TB

| **Uniprot ID** | **Entrez Gene Name from Kaneko** | **Protein Name from UNIPROT** |
| --- | --- | --- |
| P01584 | IL1B | Interleukin-1 beta |
