# Supplementary material for: Representing and querying disease networks using graph databases
Source: BioData Min. 2016 Jul 25;9:23. doi: 10.1186/s13040-016-0102-8 (PMC4960687; doi:10.1186/s13040-016-0102-8)
Supplement: Additional file 2: — ENTREZ gene name and protein names specific to the Protein nodes indicated in Figs. 1, 5, 7, 8 and 9. (DOCX 106 kb) [file 13040_2016_102_MOESM2_ESM.docx]

Additional file 2 — ENTREZ gene name and protein names specific to the Protein nodes indicated in Figures 1, 5, 7, 8 and 9

1. ENTREZ gene name and protein names specific to the Protein nodes indicated in Figures 1, 5, 7,8 and 9
2. Details for the Protein nodes indicated in Figure 1

| **UniProt ID** | **Entrez Gene Name** | **Protein Name from UNIPROT** |
| --- | --- | --- |
| P35247 | SFTPD COLEC7 PSPD SFTP4 | PULMONARY SURFACTANT-ASSOCIATED PROTEIN D (PSP-D) (SP-D) (COLLECTIN-7) (LUNG SURFACTANT PROTEIN D) |
| Q96N06 | SPATA33 C16ORF55 | SPERMATOGENESIS-ASSOCIATED PROTEIN 33 |
| P55017 | SLC12A3 NCC TSC | SOLUTE CARRIER FAMILY 12 MEMBER 3 (NA-CL COTRANSPORTER) (NCC) (NA-CL SYMPORTER) (THIAZIDE-SENSITIVE SODIUM-CHLORIDE COTRANSPORTER) |
| Q9NQ84 | GPRC5C RAIG3 PSEC0087 | G-PROTEIN COUPLED RECEPTOR FAMILY C GROUP 5 MEMBER C (RETINOIC ACID-INDUCED GENE 3 PROTEIN) (RAIG-3) |
| Q6ZUS6 | CCDC149 | COILED-COIL DOMAIN-CONTAINING PROTEIN 149 |
| P81877 | SSBP2 SSDP2 | SINGLE-STRANDED DNA-BINDING PROTEIN 2 (SEQUENCE-SPECIFIC SINGLE-STRANDED-DNA-BINDING PROTEIN 2) |
| F8WDC7 | RABL3 | RAB-LIKE PROTEIN 3 |
| Q3KP66 | C1ORF106 | UNCHARACTERIZED PROTEIN C1ORF106 |
| Q8N690 | DEFB119 DEFB120 DEFB19 DEFB20 UNQ2449/PRO5729 | BETA-DEFENSIN 119 (BETA-DEFENSIN 120) (BETA-DEFENSIN 19) (DEFB-19) (BETA-DEFENSIN 20) (DEFB-20) (DEFENSIN, BETA 119) (DEFENSIN, BETA 120) (ESC42-RELA) |
| A6NI87 | CBY3 | PROTEIN CHIBBY HOMOLOG 3 |

1. Details for the Protein nodes indicated in Figure 5

| **UniProt ID** | **Entrez Gene Name** | **Protein Name from UNIPROT** |
| --- | --- | --- |
| Q08462 | ADCY2 KIAA1060 | ADENYLATE CYCLASE TYPE 2 (EC 4.6.1.1) (ATP PYROPHOSPHATE-LYASE 2) (ADENYLATE CYCLASE TYPE II) (ADENYLYL CYCLASE 2) |
| P05113 | IL5 | INTERLEUKIN-5 (IL-5) (B-CELL DIFFERENTIATION FACTOR I) (EOSINOPHIL DIFFERENTIATION FACTOR) (T-CELL REPLACING FACTOR) (TRF) |
| P05362 | ICAM1 | INTERCELLULAR ADHESION MOLECULE 1 (ICAM-1) (MAJOR GROUP RHINOVIRUS RECEPTOR) (CD ANTIGEN CD54) |
| O95398 | RAPGEF3 CGEF1 EPAC EPAC1 | RAP GUANINE NUCLEOTIDE EXCHANGE FACTOR 3 (EXCHANGE FACTOR DIRECTLY ACTIVATED BY CAMP 1) (EXCHANGE PROTEIN DIRECTLY ACTIVATED BY CAMP 1) (EPAC 1) (RAP1 GUANINE-NUCLEOTIDE-EXCHANGE FACTOR DIRECTLY ACTIVATED BY CAMP) (CAMP-REGULATED GUANINE NUCLEOTIDE EXCHANGE FACTOR I) (CAMP-GEFI) |
| P17693 | HLA-G HLA-6.0 HLAG | HLA CLASS I HISTOCOMPATIBILITY ANTIGEN, ALPHA CHAIN G (HLA G ANTIGEN) (MHC CLASS I ANTIGEN G) |
| P01375 | TNF TNFA TNFSF2 | TUMOR NECROSIS FACTOR (CACHECTIN) (TNF-ALPHA) (TUMOR NECROSIS FACTOR LIGAND SUPERFAMILY MEMBER 2) (TNF-A) [CLEAVED INTO: TUMOR NECROSIS FACTOR, MEMBRANE FORM (N-TERMINAL FRAGMENT) (NTF); INTRACELLULAR DOMAIN 1 (ICD1); INTRACELLULAR DOMAIN 2 (ICD2); C-DOMAIN 1; C-DOMAIN 2; TUMOR NECROSIS FACTOR, SOLUBLE FORM] |

1. Details for the Protein nodes indicated in Figure 7

| **UniProt ID** | **Entrez Gene Name** | **Protein Name from UNIPROT** |
| --- | --- | --- |
| P08727 | KRT19 | KERATIN, TYPE I CYTOSKELETAL 19 (CYTOKERATIN-19) (CK-19) (KERATIN-19) (K19) |
| P05787 | KRT8 CYK8 | KERATIN, TYPE II CYTOSKELETAL 8 (CYTOKERATIN-8) (CK-8) (KERATIN-8) (K8) (TYPE-II KERATIN KB8) |
| P08729 | KRT7 SCL | KERATIN, TYPE II CYTOSKELETAL 7 (CYTOKERATIN-7) (CK-7) (KERATIN-7) (K7) (SARCOLECTIN) (TYPE-II KERATIN KB7) |
| Q99456 | KRT12 | KERATIN, TYPE I CYTOSKELETAL 12 (CYTOKERATIN-12) (CK-12) (KERATIN-12) (K12) |
| P08253 | MMP2 CLG4A | 72 KDA TYPE IV COLLAGENASE (EC 3.4.24.24) (72 KDA GELATINASE) (GELATINASE A) (MATRIX METALLOPROTEINASE-2) (MMP-2) (TBE-1) [CLEAVED INTO: PEX] |
| P09238 | MMP10 STMY2 | STROMELYSIN-2 (SL-2) (EC 3.4.24.22) (MATRIX METALLOPROTEINASE-10) (MMP-10) (TRANSIN-2) |
| P39900 | MMP12 | MACROPHAGE METALLOELASTASE |

1. Details for the Protein nodes indicated in Figure 8

| **UniProt ID** | **Entrez Gene Name** | **Protein Name from UNIPROT** |
| --- | --- | --- |
| O00327 | ARNTL BHLHE5 BMAL1 MOP3 PASD3 | ARYL HYDROCARBON RECEPTOR NUCLEAR TRANSLOCATOR-LIKE PROTEIN 1 (BASIC-HELIX-LOOP-HELIX-PAS PROTEIN MOP3) (BRAIN AND MUSCLE ARNT-LIKE 1) (CLASS E BASIC HELIX-LOOP-HELIX PROTEIN 5) (BHLHE5) (MEMBER OF PAS PROTEIN 3) (PAS DOMAIN-CONTAINING PROTEIN 3) (BHLH-PAS PROTEIN JAP3) |
| Q99814 | EPAS1 BHLHE73 HIF2A MOP2 PASD2 | ENDOTHELIAL PAS DOMAIN-CONTAINING PROTEIN 1 (EPAS-1) (BASIC-HELIX-LOOP-HELIX-PAS PROTEIN MOP2) (CLASS E BASIC HELIX-LOOP-HELIX PROTEIN 73) (BHLHE73) (HIF-1-ALPHA-LIKE FACTOR) (HLF) (HYPOXIA-INDUCIBLE FACTOR 2-ALPHA) (HIF-2-ALPHA) (HIF2-ALPHA) (MEMBER OF PAS PROTEIN 2) (PAS DOMAIN-CONTAINING PROTEIN 2) |
| P84022 | SMAD3 MADH3 | MOTHERS AGAINST DECAPENTAPLEGIC HOMOLOG 3 (MAD HOMOLOG 3) (MAD3) (MOTHERS AGAINST DPP HOMOLOG 3) (HMAD-3) (JV15-2) (SMAD FAMILY MEMBER 3) (SMAD 3) (SMAD3) (HSMAD3) |
| O15055 | PER2 KIAA0347 | PERIOD CIRCADIAN PROTEIN HOMOLOG 2 (HPER2) (CIRCADIAN CLOCK PROTEIN PERIOD 2) |
| Q92956 | TNFRSF14 HVEA HVEM UNQ329/PRO509 | TUMOR NECROSIS FACTOR RECEPTOR SUPERFAMILY MEMBER 14 (HERPES VIRUS ENTRY MEDIATOR A) (HERPESVIRUS ENTRY MEDIATOR A) (HVEA) (TUMOR NECROSIS FACTOR RECEPTOR-LIKE 2) (TR2) (CD ANTIGEN CD270) |
| P01374 | LTA TNFB TNFSF1 | LYMPHOTOXIN-ALPHA (LT-ALPHA) (TNF-BETA) (TUMOR NECROSIS FACTOR LIGAND SUPERFAMILY MEMBER 1) |
| Q16526 | CRY1 PHLL1 | CRYPTOCHROME-1 |
| Q12933 | TRAF2 TRAP3 | TNF RECEPTOR-ASSOCIATED FACTOR 2 (EC 6.3.2.-) (E3 UBIQUITIN-PROTEIN LIGASE TRAF2) (TUMOR NECROSIS FACTOR TYPE 2 RECEPTOR-ASSOCIATED PROTEIN 3) |
| P09211 | GSTP1 FAEES3 GST3 | GLUTATHIONE S-TRANSFERASE P (EC 2.5.1.18) (GST CLASS-PI) (GSTP1-1) |
| P28799 | GRN | GRANULINS (PROEPITHELIN) (PEPI) [CLEAVED INTO: ACROGRANIN (GLYCOPROTEIN OF 88 KDA) (GP88) (GLYCOPROTEIN 88) (PROGRANULIN); PARAGRANULIN; GRANULIN-1 (GRANULIN G); GRANULIN-2 (GRANULIN F); GRANULIN-3 (GRANULIN B); GRANULIN-4 (GRANULIN A); GRANULIN-5 (GRANULIN C); GRANULIN-6 (GRANULIN D); GRANULIN-7 (GRANULIN E)] |
| P13569 | CFTR ABCC7 | CYSTIC FIBROSIS TRANSMEMBRANE CONDUCTANCE REGULATOR (CFTR) (ATP-BINDING CASSETTE SUB-FAMILY C MEMBER 7) (CHANNEL CONDUCTANCE-CONTROLLING ATPASE) (EC 3.6.3.49) (CAMP-DEPENDENT CHLORIDE CHANNEL) |
| P01375 | TNF TNFA TNFSF2 | TUMOR NECROSIS FACTOR (CACHECTIN) (TNF-ALPHA) (TUMOR NECROSIS FACTOR LIGAND SUPERFAMILY MEMBER 2) (TNF-A) [CLEAVED INTO: TUMOR NECROSIS FACTOR, MEMBRANE FORM (N-TERMINAL FRAGMENT) (NTF); INTRACELLULAR DOMAIN 1 (ICD1); INTRACELLULAR DOMAIN 2 (ICD2); C-DOMAIN 1; C-DOMAIN 2; TUMOR NECROSIS FACTOR, SOLUBLE FORM] |
| Q99743 | NPAS2 BHLHE9 MOP4 PASD4 | NEURONAL PAS DOMAIN-CONTAINING PROTEIN 2 (NEURONAL PAS2) (BASIC-HELIX-LOOP-HELIX-PAS PROTEIN MOP4) (CLASS E BASIC HELIX-LOOP-HELIX PROTEIN 9) (BHLHE9) (MEMBER OF PAS PROTEIN 4) (PAS DOMAIN-CONTAINING PROTEIN 4) |
| P67870 | CSNK2B CK2N G5A | CASEIN KINASE II SUBUNIT BETA (CK II BETA) (PHOSVITIN) (PROTEIN G5A) |
| Q9UIL8 | PHF11 BCAP | PHD FINGER PROTEIN 11 (BRCA1 C-TERMINUS-ASSOCIATED PROTEIN) (RENAL CARCINOMA ANTIGEN NY-REN-34) |
| O15516 | CLOCK BHLHE8 KIAA0334 | CIRCADIAN LOCOMOTER OUTPUT CYCLES PROTEIN KAPUT (HCLOCK) (EC 2.3.1.48) (CLASS E BASIC HELIX-LOOP-HELIX PROTEIN 8) (BHLHE8) |
| Q9Y463 | DYRK1B MIRK | DUAL SPECIFICITY TYROSINE-PHOSPHORYLATION-REGULATED KINASE 1B (EC 2.7.12.1) (MINIBRAIN-RELATED KINASE) (MIRK PROTEIN KINASE) |
| Q8WYA1 | ARNTL2 BHLHE6 BMAL2 CLIF MOP9 PASD9 | ARYL HYDROCARBON RECEPTOR NUCLEAR TRANSLOCATOR-LIKE PROTEIN 2 (BASIC-HELIX-LOOP-HELIX-PAS PROTEIN MOP9) (BRAIN AND MUSCLE ARNT-LIKE 2) (CYCLE-LIKE FACTOR) (CLIF) (CLASS E BASIC HELIX-LOOP-HELIX PROTEIN 6) (BHLHE6) (MEMBER OF PAS PROTEIN 9) (PAS DOMAIN-CONTAINING PROTEIN 9) |
| E9PRB1 | ARNTL | ARYL HYDROCARBON RECEPTOR NUCLEAR TRANSLOCATOR-LIKE PROTEIN 1 (FRAGMENT) |
| E9PKG7 | ARNTL | ARYL HYDROCARBON RECEPTOR NUCLEAR TRANSLOCATOR-LIKE PROTEIN 1 (FRAGMENT) |
| E9PI92 | ARNTL | ARYL HYDROCARBON RECEPTOR NUCLEAR TRANSLOCATOR-LIKE PROTEIN 1 (FRAGMENT) |
| Q8WYA1 | ARNTL2 BHLHE6 BMAL2 CLIF MOP9 PASD9 | ARYL HYDROCARBON RECEPTOR NUCLEAR TRANSLOCATOR-LIKE PROTEIN 2 (BASIC-HELIX-LOOP-HELIX-PAS PROTEIN MOP9) (BRAIN AND MUSCLE ARNT-LIKE 2) (CYCLE-LIKE FACTOR) (CLIF) (CLASS E BASIC HELIX-LOOP-HELIX PROTEIN 6) (BHLHE6) (MEMBER OF PAS PROTEIN 9) (PAS DOMAIN-CONTAINING PROTEIN 9) |
| E9PKN1 | ARNTL | ARYL HYDROCARBON RECEPTOR NUCLEAR TRANSLOCATOR-LIKE PROTEIN 1 (FRAGMENT) |
| H0Y5R1 | ARNTL2 | ARYL HYDROCARBON RECEPTOR NUCLEAR TRANSLOCATOR-LIKE PROTEIN 2 (FRAGMENT) |
| E9PKF0 | ARNTL | ARYL HYDROCARBON RECEPTOR NUCLEAR TRANSLOCATOR-LIKE PROTEIN 1 (FRAGMENT) |
| H0YKW1 | ARNT2 | ARYL HYDROCARBON RECEPTOR NUCLEAR TRANSLOCATOR 2 (FRAGMENT) |
| E9PPV4 | ARNTL | ARYL HYDROCARBON RECEPTOR NUCLEAR TRANSLOCATOR-LIKE PROTEIN 1 (FRAGMENT) |
| E9PL54 | ARNTL | ARYL HYDROCARBON RECEPTOR NUCLEAR TRANSLOCATOR-LIKE PROTEIN 1 (FRAGMENT) |
| E9PNI4 | ARNTL | ARYL HYDROCARBON RECEPTOR NUCLEAR TRANSLOCATOR-LIKE PROTEIN 1 (FRAGMENT) |
| A6NGV6 | ARNT | ARYL HYDROCARBON RECEPTOR NUCLEAR TRANSLOCATOR |
| C9JK03 | CLOCK | CIRCADIAN LOCOMOTER OUTPUT CYCLES PROTEIN KAPUT (FRAGMENT) |
| P27540 | ARNT BHLHE2 | ARYL HYDROCARBON RECEPTOR NUCLEAR TRANSLOCATOR (ARNT PROTEIN) (CLASS E BASIC HELIX-LOOP-HELIX PROTEIN 2) (BHLHE2) (DIOXIN RECEPTOR, NUCLEAR TRANSLOCATOR) (HYPOXIA-INDUCIBLE FACTOR 1-BETA) (HIF-1-BETA) (HIF1-BETA) |
| Q9HBZ2 | ARNT2 BHLHE1 KIAA0307 | ARYL HYDROCARBON RECEPTOR NUCLEAR TRANSLOCATOR 2 (ARNT PROTEIN 2) (CLASS E BASIC HELIX-LOOP-HELIX PROTEIN 1) (BHLHE1) |
| P49674 | CSNK1E | CASEIN KINASE I ISOFORM EPSILON (CKI-EPSILON) (CKIE) (EC 2.7.11.1) |
| P56645 | PER3 GIG13 | PERIOD CIRCADIAN PROTEIN HOMOLOG 3 (HPER3) (CELL GROWTH-INHIBITING GENE 13 PROTEIN) (CIRCADIAN CLOCK PROTEIN PERIOD 3) |
| E9PD89 | PER2 | PERIOD CIRCADIAN PROTEIN HOMOLOG 2 (FRAGMENT) |
| J3QSH9 | PER1 HCG_31279 | PERIOD CIRCADIAN PROTEIN HOMOLOG 1 (PERIOD HOMOLOG 1 (DROSOPHILA), ISOFORM CRA_B) |
| Q8TAR6 | PER3 | PER3 PROTEIN (PERIOD CIRCADIAN PROTEIN HOMOLOG 3) |
| Q13191 | CBLB RNF56 NBLA00127 | E3 UBIQUITIN-PROTEIN LIGASE CBL-B (EC 6.3.2.-) (CASITAS B-LINEAGE LYMPHOMA PROTO-ONCOGENE B) (RING FINGER PROTEIN 56) (SH3-BINDING PROTEIN CBL-B) (SIGNAL TRANSDUCTION PROTEIN CBL-B) |
| Q8N2S1 | LTBP4 | LATENT-TRANSFORMING GROWTH FACTOR BETA-BINDING PROTEIN 4 (LTBP-4) |
| Q99750 | MDFI | MYOD FAMILY INHIBITOR (MYOGENIC REPRESSOR I-MF) |
| Q9BQ66 | KRTAP4-12 KAP4.12 KRTAP4.12 | KERATIN-ASSOCIATED PROTEIN 4-12 (KERATIN-ASSOCIATED PROTEIN 4.12) (ULTRAHIGH SULFUR KERATIN-ASSOCIATED PROTEIN 4.12) |
| O15162 | PLSCR1 | PHOSPHOLIPID SCRAMBLASE 1 (PL SCRAMBLASE 1) (CA(2+)-DEPENDENT PHOSPHOLIPID SCRAMBLASE 1) (ERYTHROCYTE PHOSPHOLIPID SCRAMBLASE) (MMTRA1B) |
| Q49AN0 | CRY2 KIAA0658 | CRYPTOCHROME-2 |
| H0YHT0 | CRY1 | CRYPTOCHROME-1 (FRAGMENT) |
| O14503 | BHLHE40 BHLHB2 DEC1 SHARP2 STRA13 | CLASS E BASIC HELIX-LOOP-HELIX PROTEIN 40 (BHLHE40) (CLASS B BASIC HELIX-LOOP-HELIX PROTEIN 2) (BHLHB2) (DIFFERENTIALLY EXPRESSED IN CHONDROCYTES PROTEIN 1) (DEC1) (ENHANCER-OF-SPLIT AND HAIRY-RELATED PROTEIN 2) (SHARP-2) (STIMULATED BY RETINOIC ACID GENE 13 PROTEIN) |
| P51449 | RORC NR1F3 RORG RZRG | NUCLEAR RECEPTOR ROR-GAMMA (NUCLEAR RECEPTOR RZR-GAMMA) (NUCLEAR RECEPTOR SUBFAMILY 1 GROUP F MEMBER 3) (RAR-RELATED ORPHAN RECEPTOR C) (RETINOID-RELATED ORPHAN RECEPTOR-GAMMA) |
| Q02878 | RPL6 TXREB1 | 60S RIBOSOMAL PROTEIN L6 (NEOPLASM-RELATED PROTEIN C140) (TAX-RESPONSIVE ENHANCER ELEMENT-BINDING PROTEIN 107) (TAXREB107) |
| H7C080 | NPAS2 | NEURONAL PAS DOMAIN-CONTAINING PROTEIN 2 (FRAGMENT) |
| H7C0J4 | NPAS2 | NEURONAL PAS DOMAIN-CONTAINING PROTEIN 2 (FRAGMENT) |
| H7C0Z2 | NPAS2 | NEURONAL PAS DOMAIN-CONTAINING PROTEIN 2 (FRAGMENT) |
| H7BZY5 | NPAS2 | NEURONAL PAS DOMAIN-CONTAINING PROTEIN 2 (FRAGMENT) |
| H7BZA3 | NPAS2 | NEURONAL PAS DOMAIN-CONTAINING PROTEIN 2 (FRAGMENT) |
| P62318 | SNRPD3 | SMALL NUCLEAR RIBONUCLEOPROTEIN SM D3 (SM-D3) (SNRNP CORE PROTEIN D3) |

1. Details for the Protein nodes indicated in Figure 9a

| **UniProt ID** | **Entrez Gene Name** | **Protein Name from UNIPROT** |
| --- | --- | --- |
| O15516 | CLOCK BHLHE8 KIAA0334 | CIRCADIAN LOCOMOTER OUTPUT CYCLES PROTEIN KAPUT (HCLOCK) (EC 2.3.1.48) (CLASS E BASIC HELIX-LOOP-HELIX PROTEIN 8) (BHLHE8) |
| C9JK03 | CLOCK | CIRCADIAN LOCOMOTER OUTPUT CYCLES PROTEIN KAPUT (FRAGMENT) |
| Q99743 | NPAS2 BHLHE9 MOP4 PASD4 | NEURONAL PAS DOMAIN-CONTAINING PROTEIN 2 (NEURONAL PAS2) (BASIC-HELIX-LOOP-HELIX-PAS PROTEIN MOP4) (CLASS E BASIC HELIX-LOOP-HELIX PROTEIN 9) (BHLHE9) (MEMBER OF PAS PROTEIN 4) (PAS DOMAIN-CONTAINING PROTEIN 4) |
| O00327 | ARNTL BHLHE5 BMAL1 MOP3 PASD3 | ARYL HYDROCARBON RECEPTOR NUCLEAR TRANSLOCATOR-LIKE PROTEIN 1 (BASIC-HELIX-LOOP-HELIX-PAS PROTEIN MOP3) (BRAIN AND MUSCLE ARNT-LIKE 1) (CLASS E BASIC HELIX-LOOP-HELIX PROTEIN 5) (BHLHE5) (MEMBER OF PAS PROTEIN 3) (PAS DOMAIN-CONTAINING PROTEIN 3) (BHLH-PAS PROTEIN JAP3) |
| O15055 | PER2 KIAA0347 | PERIOD CIRCADIAN PROTEIN HOMOLOG 2 (HPER2) (CIRCADIAN CLOCK PROTEIN PERIOD 2) |
| O15534 | PER1 KIAA0482 PER RIGUI | PERIOD CIRCADIAN PROTEIN HOMOLOG 1 (HPER1) (CIRCADIAN CLOCK PROTEIN PERIOD 1) (CIRCADIAN PACEMAKER PROTEIN RIGUI) |
| Q16526 | CRY1 PHLL1 | CRYPTOCHROME-1 |
| Q49AN0 | CRY2 KIAA0658 | CRYPTOCHROME-2 |
| Q9Y463 | DYRK1B MIRK | DUAL SPECIFICITY TYROSINE-PHOSPHORYLATION-REGULATED KINASE 1B (EC 2.7.12.1) (MINIBRAIN-RELATED KINASE) (MIRK PROTEIN KINASE) |
| P08238 | HSP90AB1 HSP90B HSPC2 HSPCB | HEAT SHOCK PROTEIN HSP 90-BETA (HSP 90) (HEAT SHOCK 84 KDA) (HSP 84) (HSP84) |
| P25054 | APC DP2.5 | ADENOMATOUS POLYPOSIS COLI PROTEIN (PROTEIN APC) (DELETED IN POLYPOSIS 2.5) |
| Q99814 | EPAS1 BHLHE73 HIF2A MOP2 PASD2 | ENDOTHELIAL PAS DOMAIN-CONTAINING PROTEIN 1 (EPAS-1) (BASIC-HELIX-LOOP-HELIX-PAS PROTEIN MOP2) (CLASS E BASIC HELIX-LOOP-HELIX PROTEIN 73) (BHLHE73) (HIF-1-ALPHA-LIKE FACTOR) (HLF) (HYPOXIA-INDUCIBLE FACTOR 2-ALPHA) (HIF-2-ALPHA) (HIF2-ALPHA) (MEMBER OF PAS PROTEIN 2) (PAS DOMAIN-CONTAINING PROTEIN 2) |
| P49674 | CSNK1E | CASEIN KINASE I ISOFORM EPSILON (CKI-EPSILON) (CKIE) (EC 2.7.11.1) |
| O14503 | BHLHE40 BHLHB2 DEC1 SHARP2 STRA13 | CLASS E BASIC HELIX-LOOP-HELIX PROTEIN 40 (BHLHE40) (CLASS B BASIC HELIX-LOOP-HELIX PROTEIN 2) (BHLHB2) (DIFFERENTIALLY EXPRESSED IN CHONDROCYTES PROTEIN 1) (DEC1) (ENHANCER-OF-SPLIT AND HAIRY-RELATED PROTEIN 2) (SHARP-2) (STIMULATED BY RETINOIC ACID GENE 13 PROTEIN) |
| Q00987 | MDM2 | E3 UBIQUITIN-PROTEIN LIGASE MDM2 (EC 6.3.2.-) (DOUBLE MINUTE 2 PROTEIN) (HDM2) (ONCOPROTEIN MDM2) (P53-BINDING PROTEIN MDM2) |
| Q9Y6Q9 | NCOA3 AIB1 BHLHE42 RAC3 TRAM1 | NUCLEAR RECEPTOR COACTIVATOR 3 (NCOA-3) (EC 2.3.1.48) (ACTR) (AMPLIFIED IN BREAST CANCER 1 PROTEIN) (AIB-1) (CBP-INTERACTING PROTEIN) (PCIP) (CLASS E BASIC HELIX-LOOP-HELIX PROTEIN 42) (BHLHE42) (RECEPTOR-ASSOCIATED COACTIVATOR 3) (RAC-3) (STEROID RECEPTOR COACTIVATOR PROTEIN 3) (SRC-3) (THYROID HORMONE RECEPTOR ACTIVATOR MOLECULE 1) (TRAM-1) |
| J3QSH9 | PER1 HCG_31279 | PERIOD CIRCADIAN PROTEIN HOMOLOG 1 (PERIOD HOMOLOG 1 (DROSOPHILA), ISOFORM CRA_B) |
| Q8TAR6 | PER3 | PER3 PROTEIN (PERIOD CIRCADIAN PROTEIN HOMOLOG 3 |

1. Details for the Protein nodes indicated in Figure 9b

| **UniProt ID** | **Entrez Gene Name** | **Protein Name from UNIPROT** |
| --- | --- | --- |
| P20393 | NR1D1 EAR1 HREV THRAL | NUCLEAR RECEPTOR SUBFAMILY 1 GROUP D MEMBER 1 (REV-ERBA-ALPHA) (V-ERBA-RELATED PROTEIN 1) (EAR-1) |
| Q99743 | NPAS2 BHLHE9 MOP4 PASD4 | NEURONAL PAS DOMAIN-CONTAINING PROTEIN 2 (NEURONAL PAS2) (BASIC-HELIX-LOOP-HELIX-PAS PROTEIN MOP4) (CLASS E BASIC HELIX-LOOP-HELIX PROTEIN 9) (BHLHE9) (MEMBER OF PAS PROTEIN 4) (PAS DOMAIN-CONTAINING PROTEIN 4) |
| O00327 | ARNTL BHLHE5 BMAL1 MOP3 PASD3 | ARYL HYDROCARBON RECEPTOR NUCLEAR TRANSLOCATOR-LIKE PROTEIN 1 (BASIC-HELIX-LOOP-HELIX-PAS PROTEIN MOP3) (BRAIN AND MUSCLE ARNT-LIKE 1) (CLASS E BASIC HELIX-LOOP-HELIX PROTEIN 5) (BHLHE5) (MEMBER OF PAS PROTEIN 3) (PAS DOMAIN-CONTAINING PROTEIN 3) (BHLH-PAS PROTEIN JAP3) |
| F8VVY8 | VDR | VITAMIN D3 RECEPTOR (FRAGMENT) |
| P51449 | RORC NR1F3 RORG RZRG | NUCLEAR RECEPTOR ROR-GAMMA (NUCLEAR RECEPTOR RZR-GAMMA) (NUCLEAR RECEPTOR SUBFAMILY 1 GROUP F MEMBER 3) (RAR-RELATED ORPHAN RECEPTOR C) (RETINOID-RELATED ORPHAN RECEPTOR-GAMMA) |
| O14503 | BHLHE40 BHLHB2 DEC1 SHARP2 STRA13 | CLASS E BASIC HELIX-LOOP-HELIX PROTEIN 40 (BHLHE40) (CLASS B BASIC HELIX-LOOP-HELIX PROTEIN 2) (BHLHB2) (DIFFERENTIALLY EXPRESSED IN CHONDROCYTES PROTEIN 1) (DEC1) (ENHANCER-OF-SPLIT AND HAIRY-RELATED PROTEIN 2) (SHARP-2) (STIMULATED BY RETINOIC ACID GENE 13 PROTEIN) |
| Q14995 | NR1D2 | NUCLEAR RECEPTOR SUBFAMILY 1 GROUP D MEMBER 2 (ORPHAN NUCLEAR HORMONE RECEPTOR BD73) (REV-ERB ALPHA-RELATED RECEPTOR) (RVR) (REV-ERB-BETA) (V-ERBA-RELATED PROTEIN 1-RELATED) (EAR-1R) |
| B9VVT8 | HNF4ALPHA HNF4A | HNF4ALPHA10/11/12 (HEPATOCYTE NUCLEAR FACTOR 4-ALPHA) (FRAGMENT) |
| F8VPF8 | VDR | VITAMIN D3 RECEPTOR (FRAGMENT) |
| Q9Y463 | DYRK1B MIRK | DUAL SPECIFICITY TYROSINE-PHOSPHORYLATION-REGULATED KINASE 1B (EC 2.7.12.1) (MINIBRAIN-RELATED KINASE) (MIRK PROTEIN KINASE) |
