# Supplementary material for: Representing and querying disease networks using graph databases
Source: BioData Min. 2016 Jul 25;9:23. doi: 10.1186/s13040-016-0102-8 (PMC4960687; doi:10.1186/s13040-016-0102-8)
Supplement: Additional file 3: — Brief introduction to Cypher query language; for more details on the Cypher language, the reader is referred to the Neo4j website: http://neo4j.com/. (DOCX 17 kb) [file 13040_2016_102_MOESM3_ESM.docx]

Additional file 3 — Brief introduction to Cypher query language; for more details on the Cypher language, the reader is referred to the Neo4j website: <http://neo4j.com/>

Cypher is an SQL-like query language for graphs used by Neo4J NoSQL database. Cypher language is high-level and its syntax is designed to be most natural and convenient to the user. Most of the optimisation is done by the query engine that can automatically optimise the queries and indexing required to ensure adequate performance. Neo4J graphs are typed, attributed, directed multigraphs, where edges have a type and source-target nodes, can have attributes and multiple edges are allowed between the same pair of nodes. The query has the following parts:

1. MATCH clause – this specifies a graph pattern using ASCII art-like syntax. In the pattern the nodes are repressed by elements in round brackets and edges – in square brackets. The basic pattern is of the form ()-[]->() and can also be undirected, e.g. ()-[]-(). Each set of brackets can contain additional qualifiers applicable to that particular element in the form of variable_id:type_constant{key1:’value1’,key2:’value2’} Here ‘variable_id’ is a query-scope identifier given to that particular node or edge by which it can referenced in the remainder of the query. ‘type_constant’ or XXX in the case of nodes is the graph-unique class identifier for a particular set of nodes or edges and the ‘{}’ is the list of properties to match in the form of a key-value map. All of these differ qualifiers are optional and can be omitted as necessary. A branching pattern can be specified by separating multiple patterns with a comma and an additional ‘OPTIONAL MATCH’ clause can be used for parts of the pattern than may not always be present.
2. WHERE clause – is optional and allows more complex conditions for pattern to be specified, like Boolean logic operators or mathematical expressions like ‘==’,’>=’ and ‘<>’. In the WHERE clause the properties of nodes and edges can be referred as ‘variable_id.key1’, where ‘variable_id’ is the same as the one defined in the MATCH clause as key1 is the name of the property. WHERE clause can also contain calls to functions of the form ‘function_name(arg1, arg2, arg3 …)’ that can be used describe more complex qualifiers for the pattern.
3. RETURN clause – specifies what parts of the matched graph are retained as a result of the query. It is possible to return both parts of the graph or tables of values. In the latter case the behaviour is exactly the same as in SQL relation database – one combined table is returned with individual values arranged according to the relationships created as part of the query. The RETURN clause can contain additional instructions for formatting the data, in particular the ‘AS’ keyword that can bind a variable to a different name a ‘collect’ function that compiles multiple entities into a list and ‘ORDER BY’ keyword, which allows sorting of the result tables, among others.

This outline only describes the most fundamental parts of the Cypher query language, and many more options are available both as additional keywords and as in-build functions. Among the more advanced features, we would like to mention the support for collections (maps, sets and lists), string operations (regex pattern matching, splitting/concatenation), mathematical operations and path functions, which allow manipulation of matched subgraphs. For a more comprehensive introduction we would like to direct the readers to the official Neo4J Cypher guide at <http://neo4j.com/docs/stable/cypher-introduction.html>. Cypher language can also be used to dynamically alter the graph with CREATE clause, which allows new nodes and edges to be introduced by using the same pattern syntax as in MATCH clause, SET clause for changing/creating the value of an individual attribute and DELETE or REMOVE clause for deleting elements of the graph and individual attributes, respectively.
