# Supplementary material for: Representing and querying disease networks using graph databases
Source: BioData Min. 2016 Jul 25;9:23. doi: 10.1186/s13040-016-0102-8 (PMC4960687; doi:10.1186/s13040-016-0102-8)
Supplement: Additional file 4: — Information on genes related to a simple disease only, as reported in [23]. (DOCX 120 kb) [file 13040_2016_102_MOESM4_ESM.docx]

Additional file 4 — Information on genes related to a simple disease only, as reported in [23]

1. Proteins specific to Asthma only

| **Uniprot ID** | **Gene Symbol** | **Protein Name from UNIPROT** |
| --- | --- | --- |
| P84022 | SMAD3; MADH3 | MOTHERS AGAINST DECAPENTAPLEGIC HOMOLOG 3 (MAD HOMOLOG 3) (MAD3) (MOTHERS AGAINST DPP HOMOLOG 3) (HMAD-3) (JV15-2) (SMAD FAMILY MEMBER 3) (SMAD 3) (SMAD3) (HSMAD3) |
| P40763 | STAT3; APRF | SIGNAL TRANSDUCER AND ACTIVATOR OF TRANSCRIPTION 3 (ACUTE-PHASE RESPONSE FACTOR) |
| P42226 | STAT6 | SIGNAL TRANSDUCER AND ACTIVATOR OF TRANSCRIPTION 6 (IL-4 STAT) |
| P21731 | TBXA2R | THROMBOXANE A2 RECEPTOR (TXA2-R) (PROSTANOID TP RECEPTOR) |
| Q9UL17 | TBX21; TBET; TBLYM | T-BOX TRANSCRIPTION FACTOR TBX21 (T-BOX PROTEIN 21) (T-CELL-SPECIFIC T-BOX TRANSCRIPTION FACTOR T-BET) (TRANSCRIPTION FACTOR TBLYM) |
| P37231 | PPARG; NR1C3 | PEROXISOME PROLIFERATOR-ACTIVATED RECEPTOR GAMMA (PPAR-GAMMA) (NUCLEAR RECEPTOR SUBFAMILY 1 GROUP C MEMBER 3) |
| Q13258 | PTGDR | PROSTAGLANDIN D2 RECEPTOR (PGD RECEPTOR) (PGD2 RECEPTOR) (PROSTANOID DP RECEPTOR) |
| Q9Y5Y4 | PTGDR2; CRTH2; DL1R; GPR44 | PROSTAGLANDIN D2 RECEPTOR 2 (CHEMOATTRACTANT RECEPTOR-HOMOLOGOUS MOLECULE EXPRESSED ON TH2 CELLS) (G-PROTEIN COUPLED RECEPTOR 44) (CD ANTIGEN CD294) |
| P43116 | PTGER2 | PROSTAGLANDIN E2 RECEPTOR EP2 SUBTYPE (PGE RECEPTOR EP2 SUBTYPE) (PGE2 RECEPTOR EP2 SUBTYPE) (PROSTANOID EP2 RECEPTOR) |
| P43115 | PTGER3 | PROSTAGLANDIN E2 RECEPTOR EP3 SUBTYPE (PGE RECEPTOR EP3 SUBTYPE) (PGE2 RECEPTOR EP3 SUBTYPE) (PGE2-R) (PROSTANOID EP3 RECEPTOR) |
| Q92878 | RAD50 | DNA REPAIR PROTEIN RAD50 (HRAD50) (EC 3.6.-.-) |
| P35398 | RORA; NR1F1; RZRA | NUCLEAR RECEPTOR ROR-ALPHA (NUCLEAR RECEPTOR RZR-ALPHA) (NUCLEAR RECEPTOR SUBFAMILY 1 GROUP F MEMBER 1) (RAR-RELATED ORPHAN RECEPTOR A) (RETINOID-RELATED ORPHAN RECEPTOR-ALPHA) |
| P11684 | SCGB1A1; CC10; CCSP; UGB | UTEROGLOBIN (CLARA CELL PHOSPHOLIPID-BINDING PROTEIN) (CCPBP) (CLARA CELLS 10 KDA SECRETORY PROTEIN) (CC10) (SECRETOGLOBIN FAMILY 1A MEMBER 1) (URINARY PROTEIN 1) (UP-1) (UP1) (URINE PROTEIN 1) |
| Q9Y2C9 | TLR6 | TOLL-LIKE RECEPTOR 6 (CD ANTIGEN CD286) |
| P24821 | TNC; HXB | TENASCIN (TN) (CYTOTACTIN) (GMEM) (GP 150-225) (GLIOMA-ASSOCIATED-EXTRACELLULAR MATRIX ANTIGEN) (HEXABRACHION) (JI) (MYOTENDINOUS ANTIGEN) (NEURONECTIN) (TENASCIN-C) (TN-C) |
| Q969D9 | TSLP | THYMIC STROMAL LYMPHOPOIETIN |
| P15692 | VEGFA; VEGF | VASCULAR ENDOTHELIAL GROWTH FACTOR A (VEGF-A) (VASCULAR PERMEABILITY FACTOR) (VPF) |
| P24394 | IL4R; IL4RA; 582J2.1 | INTERLEUKIN-4 RECEPTOR SUBUNIT ALPHA (IL-4 RECEPTOR SUBUNIT ALPHA) (IL-4R SUBUNIT ALPHA) (IL-4R-ALPHA) (IL-4RA) (CD ANTIGEN CD124) [CLEAVED INTO: SOLUBLE INTERLEUKIN-4 RECEPTOR SUBUNIT ALPHA (SOLUBLE IL-4 RECEPTOR SUBUNIT ALPHA) (SOLUBLE IL-4R-ALPHA) (SIL4RALPHA/PROT) (IL-4-BINDING PROTEIN) (IL4-BP)] |
| P08887 | IL6R | INTERLEUKIN-6 RECEPTOR SUBUNIT ALPHA (IL-6 RECEPTOR SUBUNIT ALPHA) (IL-6R SUBUNIT ALPHA) (IL-6R-ALPHA) (IL-6RA) (IL-6R 1) (MEMBRANE GLYCOPROTEIN 80) (GP80) (CD ANTIGEN CD126) |
| O95760 | IL33; C9ORF26; IL1F11; NFHEV | "INTERLEUKIN-33 (IL-33) (INTERLEUKIN-1 FAMILY MEMBER 11) (IL-1F11) (NUCLEAR FACTOR FROM HIGH ENDOTHELIAL VENULES) (NF-HEV) [CLEAVED INTO: INTERLEUKIN-33 (95-270); INTERLEUKIN-33 (99-270); INTERLEUKIN-33 (109-270)]" |
| P05106 | ITGB3 | INTEGRIN BETA-3 (PLATELET MEMBRANE GLYCOPROTEIN IIIA) (GPIIIA) (CD ANTIGEN CD61) |
| Q14392 | LRRC32; D11S833E; GARP | LEUCINE-RICH REPEAT-CONTAINING PROTEIN 32 (GARPIN) (GLYCOPROTEIN A REPETITIONS PREDOMINANT) (GARP) |
| P10145 | CXCL8; IL8 | "INTERLEUKIN-8 (IL-8) (C-X-C MOTIF CHEMOKINE 8) (CHEMOKINE (C-X-C MOTIF) LIGAND 8) (EMOCTAKIN) (GRANULOCYTE CHEMOTACTIC PROTEIN 1) (GCP-1) (MONOCYTE-DERIVED NEUTROPHIL CHEMOTACTIC FACTOR) (MDNCF) (MONOCYTE-DERIVED NEUTROPHIL-ACTIVATING PEPTIDE) (MONAP) (NEUTROPHIL-ACTIVATING PROTEIN 1) (NAP-1) (PROTEIN 3-10C) (T-CELL CHEMOTACTIC FACTOR) [CLEAVED INTO: MDNCF-A (GCP/IL-8 PROTEIN IV) (IL8/NAP1 FORM I); INTERLEUKIN-8 ((ALA-IL-8)77) (GCP/IL-8 PROTEIN II) (IL-8(1-77)) (IL8/NAP1 FORM II) (MDNCF-B); IL-8(5-77); IL-8(6-77) ((SER-IL-8)72) (GCP/IL-8 PROTEIN I) (IL8/NAP1 FORM III) (LYMPHOCYTE-DERIVED NEUTROPHIL-ACTIVATING FACTOR) (LYNAP) (MDNCF-C) (NEUTROPHIL-ACTIVATING FACTOR) (NAF); IL-8(7-77) (GCP/IL-8 PROTEIN V) (IL8/NAP1 FORM IV); IL-8(8-77) (GCP/IL-8 PROTEIN VI) (IL8/NAP1 FORM V); IL-8(9-77) (GCP/IL-8 PROTEIN III) (IL8/NAP1 FORM VI)]" |
| P10914 | IRF1 | INTERFERON REGULATORY FACTOR 1 (IRF-1) |
| Q14116 | IL18; IGIF; IL1F4 | INTERLEUKIN-18 (IL-18) (IBOCTADEKIN) (INTERFERON GAMMA-INDUCING FACTOR) (IFN-GAMMA-INDUCING FACTOR) (INTERLEUKIN-1 GAMMA) (IL-1 GAMMA) |
| Q13478 | IL18R1; IL1RRP | INTERLEUKIN-18 RECEPTOR 1 (IL-18R-1) (IL-18R1) (CD218 ANTIGEN-LIKE FAMILY MEMBER A) (CDW218A) (IL1 RECEPTOR-RELATED PROTEIN) (IL-1RRP) (IL1R-RP) (CD ANTIGEN CD218A) |
| Q16552 | IL17A; CTLA8; IL17 | INTERLEUKIN-17A (IL-17) (IL-17A) (CYTOTOXIC T-LYMPHOCYTE-ASSOCIATED ANTIGEN 8) (CTLA-8) |
| Q96PD4 | IL17F | INTERLEUKIN-17F (IL-17F) (CYTOKINE ML-1) |
| Q01638 | IL1RL1; DER4; ST2 T1 | INTERLEUKIN-1 RECEPTOR-LIKE 1 (PROTEIN ST2) |
| P14784 | IL2RB | INTERLEUKIN-2 RECEPTOR SUBUNIT BETA (IL-2 RECEPTOR SUBUNIT BETA) (IL-2R SUBUNIT BETA) (IL-2RB) (HIGH AFFINITY IL-2 RECEPTOR SUBUNIT BETA) (P70-75) (P75) (CD ANTIGEN CD122) |
| P18510 | IL1RN; IL1F3; IL1RA | INTERLEUKIN-1 RECEPTOR ANTAGONIST PROTEIN (IL-1RN) (IL-1RA) (IRAP) (ICIL-1RA) (IL1 INHIBITOR) (ANAKINRA) |
| Q8N138 | ORMDL3 | ORM1-LIKE PROTEIN 3 |
| P15559 | NQO1; DIA4; NMOR1 | NAD(P)H DEHYDROGENASE [QUINONE] 1 (EC 1.6.5.2) (AZOREDUCTASE) (DT-DIAPHORASE) (DTD) (MENADIONE REDUCTASE) (NAD(P)H:QUINONE OXIDOREDUCTASE 1) (PHYLLOQUINONE REDUCTASE) (QUINONE REDUCTASE 1) (QR1) |
| Q6W5P4 | NPSR1; GPR154; GPRA; PGR14 | NEUROPEPTIDE S RECEPTOR (G-PROTEIN COUPLED RECEPTOR 154) (G-PROTEIN COUPLED RECEPTOR PGR14) (G-PROTEIN COUPLED RECEPTOR FOR ASTHMA SUSCEPTIBILITY) |
| Q13093 | PLA2G7; PAFAH | PLATELET-ACTIVATING FACTOR ACETYLHYDROLASE (PAF ACETYLHYDROLASE) (EC 3.1.1.47) (1-ALKYL-2-ACETYLGLYCEROPHOSPHOCHOLINE ESTERASE) (2-ACETYL-1-ALKYLGLYCEROPHOSPHOCHOLINE ESTERASE) (GROUP-VIIA PHOSPHOLIPASE A2) (GVIIA-PLA2) (LDL-ASSOCIATED PHOSPHOLIPASE A2) (LDL-PLA(2)) (PAF 2-ACYLHYDROLASE) |
| Q9UIL8 | PHF11; BCAP | PHD FINGER PROTEIN 11 (BRCA1 C-TERMINUS-ASSOCIATED PROTEIN) (RENAL CARCINOMA ANTIGEN NY-REN-34) |
| Q96FM1 | PGAP3; CAB2; PERLD1; UNQ546/PRO1100 | POST-GPI ATTACHMENT TO PROTEINS FACTOR 3 (COS16 HOMOLOG) (HCOS16) (GENE COAMPLIFIED WITH ERBB2 PROTEIN) (PER1-LIKE DOMAIN-CONTAINING PROTEIN 1) |
| P05121 | SERPINE1; PAI1; PLANH1 | PLASMINOGEN ACTIVATOR INHIBITOR 1 (PAI) (PAI-1) (ENDOTHELIAL PLASMINOGEN ACTIVATOR INHIBITOR) (SERPIN E1) |
| Q01362 | MS4A2; APY; FCER1B; IGER | HIGH AFFINITY IMMUNOGLOBULIN EPSILON RECEPTOR SUBUNIT BETA (FCERI) (FC EPSILON RECEPTOR I BETA-CHAIN) (IGE FC RECEPTOR SUBUNIT BETA) (MEMBRANE-SPANNING 4-DOMAINS SUBFAMILY A MEMBER 2) |
| Q16873 | LTC4S | LEUKOTRIENE C4 SYNTHASE (LTC4 SYNTHASE) (EC 4.4.1.20) (LEUKOTRIENE-C(4) SYNTHASE) |
| P09960 | LTA4H; LTA4 | LEUKOTRIENE A-4 HYDROLASE (LTA-4 HYDROLASE) (EC 3.3.2.6) (LEUKOTRIENE A(4) HYDROLASE) |
| P01374 | LTA TNFB TNFSF1 | LYMPHOTOXIN-ALPHA (LT-ALPHA) (TNF-BETA) (TUMOR NECROSIS FACTOR LIGAND SUPERFAMILY MEMBER 1) |
| P29475 | NOS1 | NITRIC OXIDE SYNTHASE, BRAIN (EC 1.14.13.39) (CONSTITUTIVE NOS) (NC-NOS) (NOS TYPE I) (NEURONAL NOS) (N-NOS) (NNOS) (PEPTIDYL-CYSTEINE S-NITROSYLASE NOS1) (BNOS) |
| P11245 | NAT2 AAC2 | ARYLAMINE N-ACETYLTRANSFERASE 2 (EC 2.3.1.5) (ARYLAMIDE ACETYLASE 2) (N-ACETYLTRANSFERASE TYPE 2) (NAT-2) (POLYMORPHIC ARYLAMINE N-ACETYLTRANSFERASE) (PNAT) |
| Q15746 | MYLK MLCK MLCK1 MYLK1 | MYOSIN LIGHT CHAIN KINASE, SMOOTH MUSCLE (MLCK) (SMMLCK) (EC 2.7.11.18) (KINASE-RELATED PROTEIN) (KRP) (TELOKIN) [CLEAVED INTO: MYOSIN LIGHT CHAIN KINASE, SMOOTH MUSCLE, DEGLUTAMYLATED FORM] |
| Q8TAX7 | MUC7 MG2 | MUCIN-7 (MUC-7) (APO-MG2) (SALIVARY MUCIN-7) |
| Q6P3S1 | DENND1B C1ORF218 FAM31B | DENN DOMAIN-CONTAINING PROTEIN 1B (CONNECDENN 2) (PROTEIN FAM31B) |
| Q8N608 | DPP10 DPRP3 KIAA1492 | INACTIVE DIPEPTIDYL PEPTIDASE 10 (DIPEPTIDYL PEPTIDASE IV-RELATED PROTEIN 3) (DPRP-3) (DIPEPTIDYL PEPTIDASE X) (DPP X) (DIPEPTIDYL PEPTIDASE-LIKE PROTEIN 2) (DPL2) |
| P06734 | FCER2 CD23A CLEC4J FCE2 IGEBF | "LOW AFFINITY IMMUNOGLOBULIN EPSILON FC RECEPTOR (BLAST-2) (C-TYPE LECTIN DOMAIN FAMILY 4 MEMBER J) (FC-EPSILON-RII) (IMMUNOGLOBULIN E-BINDING FACTOR) (LYMPHOCYTE IGE RECEPTOR) (CD ANTIGEN CD23) [CLEAVED INTO: LOW AFFINITY IMMUNOGLOBULIN EPSILON FC RECEPTOR MEMBRANE-BOUND FORM; LOW AFFINITY IMMUNOGLOBULIN EPSILON FC RECEPTOR SOLUBLE FORM]" |
| Q9Y271 | CYSLTR1 CYSLT1 | CYSTEINYL LEUKOTRIENE RECEPTOR 1 (CYSLTR1) (CYSTEINYL LEUKOTRIENE D4 RECEPTOR) (LTD4 RECEPTOR) (G-PROTEIN COUPLED RECEPTOR HG55) (HMTMF81) |
| Q9NS75 | CYSLTR2 CYSLT2 CYSLT2R PSEC0146 | CYSTEINYL LEUKOTRIENE RECEPTOR 2 (CYSLTR2) (G-PROTEIN COUPLED RECEPTOR GPCR21) (HGPCR21) (G-PROTEIN COUPLED RECEPTOR HG57) (HPN321) |
| P60022 | DEFB1 BD1 HBD1 | BETA-DEFENSIN 1 (BD-1) (HBD-1) (DEFENSIN, BETA 1) |
| Q9BZP6 | CHIA | ACIDIC MAMMALIAN CHITINASE (AMCASE) (EC 3.2.1.14) (LUNG-SPECIFIC PROTEIN TSA1902) |
| P23946 | CMA1 CYH CYM | CHYMASE (EC 3.4.21.39) (ALPHA-CHYMASE) (MAST CELL PROTEASE I) |
| P35354 | PTGS2 COX2 | PROSTAGLANDIN G/H SYNTHASE 2 (EC 1.14.99.1) (CYCLOOXYGENASE-2) (COX-2) (PHS II) (PROSTAGLANDIN H2 SYNTHASE 2) (PGH SYNTHASE 2) (PGHS-2) (PROSTAGLANDIN-ENDOPEROXIDE SYNTHASE 2) |
| O95727 | CRTAM | CYTOTOXIC AND REGULATORY T-CELL MOLECULE (CLASS-I MHC-RESTRICTED T-CELL-ASSOCIATED MOLECULE) (CD ANTIGEN CD355) |
| P51677 | CCR3 CMKBR3 | C-C CHEMOKINE RECEPTOR TYPE 3 (C-C CKR-3) (CC-CKR-3) (CCR-3) (CCR3) (CKR3) (EOSINOPHIL EOTAXIN RECEPTOR) (CD ANTIGEN CD193) |
| P08571 | CD14 | "MONOCYTE DIFFERENTIATION ANTIGEN CD14 (MYELOID CELL-SPECIFIC LEUCINE-RICH GLYCOPROTEIN) (CD ANTIGEN CD14) [CLEAVED INTO: MONOCYTE DIFFERENTIATION ANTIGEN CD14, URINARY FORM; MONOCYTE DIFFERENTIATION ANTIGEN CD14, MEMBRANE-BOUND FORM]" |
| P13569 | CFTR ABCC7 | CYSTIC FIBROSIS TRANSMEMBRANE CONDUCTANCE REGULATOR (CFTR) (ATP-BINDING CASSETTE SUB-FAMILY C MEMBER 7) (CHANNEL CONDUCTANCE-CONTROLLING ATPASE) (EC 3.6.3.49) (CAMP-DEPENDENT CHLORIDE CHANNEL) |
| P36222 | CHI3L1 | CHITINASE-3-LIKE PROTEIN 1 (39 KDA SYNOVIAL PROTEIN) (CARTILAGE GLYCOPROTEIN 39) (CGP-39) (GP-39) (HCGP-39) (YKL-40) |
| P40933 | IL15 | INTERLEUKIN-15 (IL-15) |
| P20036 | HLA-DPA1 HLA-DP1A HLASB | HLA CLASS II HISTOCOMPATIBILITY ANTIGEN, DP ALPHA 1 CHAIN (DP(W3)) (DP(W4)) (HLA-SB ALPHA CHAIN) (MHC CLASS II DP3-ALPHA) (MHC CLASS II DPA1) |
| Q96D42 | HAVCR1 KIM1 TIM1 TIMD1 | HEPATITIS A VIRUS CELLULAR RECEPTOR 1 (HAVCR-1) (KIDNEY INJURY MOLECULE 1) (KIM-1) (T-CELL IMMUNOGLOBULIN AND MUCIN DOMAIN-CONTAINING PROTEIN 1) (TIMD-1) (T-CELL IMMUNOGLOBULIN MUCIN RECEPTOR 1) (TIM) (TIM-1) (T-CELL MEMBRANE PROTEIN 1) |
| Q8TDQ0 | HAVCR2 TIM3 TIMD3 | HEPATITIS A VIRUS CELLULAR RECEPTOR 2 (HAVCR-2) (T-CELL IMMUNOGLOBULIN AND MUCIN DOMAIN-CONTAINING PROTEIN 3) (TIMD-3) (T-CELL IMMUNOGLOBULIN MUCIN RECEPTOR 3) (TIM-3) (T-CELL MEMBRANE PROTEIN 3) |
| Q8TAX9 | GSDMB GSDML PP4052 PRO2521 | GASDERMIN-B (GASDERMIN-LIKE PROTEIN) |
| P20930 | FLG | FILAGGRIN |
| P09110 | ACAA1 ACAA PTHIO | 3-KETOACYL-COA THIOLASE, PEROXISOMAL (EC 2.3.1.16) (ACETYL-COA ACYLTRANSFERASE) (BETA-KETOTHIOLASE) (PEROXISOMAL 3-OXOACYL-COA THIOLASE) |
| P41597 | CCR2 CMKBR2 | C-C CHEMOKINE RECEPTOR TYPE 2 (C-C CKR-2) (CC-CKR-2) (CCR-2) (CCR2) (MONOCYTE CHEMOATTRACTANT PROTEIN 1 RECEPTOR) (MCP-1-R) (CD ANTIGEN CD192) |
| O00175 | CCL24 MPIF2 SCYA24 | C-C MOTIF CHEMOKINE 24 (CK-BETA-6) (EOSINOPHIL CHEMOTACTIC PROTEIN 2) (EOTAXIN-2) (MYELOID PROGENITOR INHIBITORY FACTOR 2) (MPIF-2) (SMALL-INDUCIBLE CYTOKINE A24) |
| P51671 | CCL11 SCYA11 | EOTAXIN (C-C MOTIF CHEMOKINE 11) (EOSINOPHIL CHEMOTACTIC PROTEIN) (SMALL-INDUCIBLE CYTOKINE A11) |
| P01031 | C5 CPAMD4 | "COMPLEMENT C5 (C3 AND PZP-LIKE ALPHA-2-MACROGLOBULIN DOMAIN-CONTAINING PROTEIN 4) [CLEAVED INTO: COMPLEMENT C5 BETA CHAIN; COMPLEMENT C5 ALPHA CHAIN; C5A ANAPHYLATOXIN; COMPLEMENT C5 ALPHA' CHAIN]" |
| P01024 | C3 CPAMD1 | "COMPLEMENT C3 (C3 AND PZP-LIKE ALPHA-2-MACROGLOBULIN DOMAIN-CONTAINING PROTEIN 1) [CLEAVED INTO: COMPLEMENT C3 BETA CHAIN; C3-BETA-C (C3BC); COMPLEMENT C3 ALPHA CHAIN; C3A ANAPHYLATOXIN; ACYLATION STIMULATING PROTEIN (ASP) (C3ADESARG); COMPLEMENT C3B ALPHA' CHAIN; COMPLEMENT C3C ALPHA' CHAIN FRAGMENT 1; COMPLEMENT C3DG FRAGMENT; COMPLEMENT C3G FRAGMENT; COMPLEMENT C3D FRAGMENT; COMPLEMENT C3F FRAGMENT; COMPLEMENT C3C ALPHA' CHAIN FRAGMENT 2]" |
| P23560 | BDNF | BRAIN-DERIVED NEUROTROPHIC FACTOR (BDNF) (ABRINEURIN) |
| Q5TC12 | ATPAF1 ATP11 | ATP SYNTHASE MITOCHONDRIAL F1 COMPLEX ASSEMBLY FACTOR 1 (ATP11 HOMOLOG) |
| Q9H1Y0 | ATG5 APG5L ASP | AUTOPHAGY PROTEIN 5 (APG5-LIKE) (APOPTOSIS-SPECIFIC PROTEIN) |
| P78540 | ARG2 | ARGINASE-2, MITOCHONDRIAL (EC 3.5.3.1) (KIDNEY-TYPE ARGINASE) (NON-HEPATIC ARGINASE) (TYPE II ARGINASE) |
| P05089 | ARG1 | ARGINASE-1 (EC 3.5.3.1) (LIVER-TYPE ARGINASE) (TYPE I ARGINASE) |
| P20292 | ALOX5AP FLAP | ARACHIDONATE 5-LIPOXYGENASE-ACTIVATING PROTEIN (FLAP) (MK-886-BINDING PROTEIN) |

1. Proteins specific COPD only

| Uniprot ID | Gene Symbol | Protein Name from UNIPROT |
| --- | --- | --- |
| P18887 | XRCC1 | DNA REPAIR PROTEIN XRCC1 (X-RAY REPAIR CROSS-COMPLEMENTING PROTEIN 1) |
| P08294 | SOD3 | EXTRACELLULAR SUPEROXIDE DISMUTASE [CU-ZN] (EC-SOD) (EC 1.15.1.1) |
| Q8IXJ6 | SIRT2 SIR2L SIR2L2 | NAD-DEPENDENT PROTEIN DEACETYLASE SIRTUIN-2 (EC 3.5.1.-) (REGULATORY PROTEIN SIR2 HOMOLOG 2) (SIR2-LIKE PROTEIN 2) |
| P35247 | SFTPD COLEC7 PSPD SFTP4 | PULMONARY SURFACTANT-ASSOCIATED PROTEIN D (PSP-D) (SP-D) (COLLECTIN-7) (LUNG SURFACTANT PROTEIN D) |
| P07988 | SFTPB SFTP3 | PULMONARY SURFACTANT-ASSOCIATED PROTEIN B (SP-B) (18 KDA PULMONARY-SURFACTANT PROTEIN) (6 KDA PROTEIN) (PULMONARY SURFACTANT-ASSOCIATED PROTEOLIPID SPL(PHE)) |
| P07093 | SERPINE2 PI7 PN1 | GLIA-DERIVED NEXIN (GDN) (PEPTIDASE INHIBITOR 7) (PI-7) (PROTEASE NEXIN 1) (PN-1) (PROTEASE NEXIN I) (SERPIN E2) |
| P01009 | SERPINA1 AAT PI PRO0684 PRO2209 | ALPHA-1-ANTITRYPSIN (ALPHA-1 PROTEASE INHIBITOR) (ALPHA-1-ANTIPROTEINASE) (SERPIN A1) [CLEAVED INTO: SHORT PEPTIDE FROM AAT (SPAAT)] |
| Q9UMR5 | PPT2 | LYSOSOMAL THIOESTERASE PPT2 (PPT-2) (EC 3.1.2.-) (S-THIOESTERASE G14) |
| Q08499 | PDE4D DPDE3 | CAMP-SPECIFIC 3',5'-CYCLIC PHOSPHODIESTERASE 4D (EC 3.1.4.53) (DPDE3) (PDE43) |
| Q9HBA0 | TRPV4 VRL2 VROAC | TRANSIENT RECEPTOR POTENTIAL CATION CHANNEL SUBFAMILY V MEMBER 4 (TRPV4) (OSM-9-LIKE TRP CHANNEL 4) (OTRPC4) (TRANSIENT RECEPTOR POTENTIAL PROTEIN 12) (TRP12) (VANILLOID RECEPTOR-LIKE CHANNEL 2) (VANILLOID RECEPTOR-LIKE PROTEIN 2) (VRL-2) (VANILLOID RECEPTOR-RELATED OSMOTICALLY-ACTIVATED CHANNEL) (VR-OAC) |
| P04637 | TP53 P53 | CELLULAR TUMOR ANTIGEN P53 (ANTIGEN NY-CO-13) (PHOSPHOPROTEIN P53) (TUMOR SUPPRESSOR P53) |
| Q9HBL0 | TNS1 TNS | TENSIN-1 |
| Q6ZMP0 | THSD4 UNQ9334/PRO34005 | THROMBOSPONDIN TYPE-1 DOMAIN-CONTAINING PROTEIN 4 (A DISINTEGRIN AND METALLOPROTEINASE WITH THROMBOSPONDIN MOTIFS-LIKE PROTEIN 6) (ADAMTS-LIKE PROTEIN 6) (ADAMTSL-6) |
| P42224 | STAT1 | SIGNAL TRANSDUCER AND ACTIVATOR OF TRANSCRIPTION 1-ALPHA/BETA (TRANSCRIPTION FACTOR ISGF-3 COMPONENTS P91/P84) |
| P05231 | IL6 IFNB2 | INTERLEUKIN-6 (IL-6) (B-CELL STIMULATORY FACTOR 2) (BSF-2) (CTL DIFFERENTIATION FACTOR) (CDF) (HYBRIDOMA GROWTH FACTOR) (INTERFERON BETA-2) (IFN-BETA-2) |
| Q96CB8 | INTS12 PHF22 SBBI22 | INTEGRATOR COMPLEX SUBUNIT 12 (INT12) (PHD FINGER PROTEIN 22) |
| P48200 | IREB2 | IRON-RESPONSIVE ELEMENT-BINDING PROTEIN 2 (IRE-BP 2) (IRON REGULATORY PROTEIN 2) (IRP2) |
| P41159 | LEP OB OBS | LEPTIN (OBESE PROTEIN) (OBESITY FACTOR) |
| Q13639 | HTR4 | 5-HYDROXYTRYPTAMINE RECEPTOR 4 (5-HT-4) (5-HT4) (SEROTONIN RECEPTOR 4) |
| P21757 | MSR1 SCARA1 | MACROPHAGE SCAVENGER RECEPTOR TYPES I AND II (MACROPHAGE ACETYLATED LDL RECEPTOR I AND II) (SCAVENGER RECEPTOR CLASS A MEMBER 1) (CD ANTIGEN CD204) |
| Q15653 | NFKBIB IKBB TRIP9 | NF-KAPPA-B INHIBITOR BETA (NF-KAPPA-BIB) (I-KAPPA-B-BETA) (IKB-B) (IKB-BETA) (IKAPPABBETA) (THYROID RECEPTOR-INTERACTING PROTEIN 9) (TR-INTERACTING PROTEIN 9) (TRIP-9) |
| Q6UXI9 | NPNT EGFL6L POEM UNQ295/PRO334 | NEPHRONECTIN (PREOSTEOBLAST EGF-LIKE REPEAT PROTEIN WITH MAM DOMAIN) (PROTEIN EGFL6-LIKE) |
| P33527 | ABCC1 MRP MRP1 | MULTIDRUG RESISTANCE-ASSOCIATED PROTEIN 1 (ATP-BINDING CASSETTE SUB-FAMILY C MEMBER 1) (LEUKOTRIENE C(4) TRANSPORTER) (LTC4 TRANSPORTER) |
| P32297 | CHRNA3 NACHRA3 | NEURONAL ACETYLCHOLINE RECEPTOR SUBUNIT ALPHA-3 |
| P30532 | CHRNA5 NACHRA5 | NEURONAL ACETYLCHOLINE RECEPTOR SUBUNIT ALPHA-5 |
| Q15109 | AGER RAGE | ADVANCED GLYCOSYLATION END PRODUCT-SPECIFIC RECEPTOR (RECEPTOR FOR ADVANCED GLYCOSYLATION END PRODUCTS) |
| Q96G01 | BICD1 | PROTEIN BICAUDAL D HOMOLOG 1 (BIC-D 1) |
| Q9H013 | ADAM19 MLTNB FKSG34 | DISINTEGRIN AND METALLOPROTEINASE DOMAIN-CONTAINING PROTEIN 19 (ADAM 19) (EC 3.4.24.-) (MELTRIN-BETA) (METALLOPROTEASE AND DISINTEGRIN DENDRITIC ANTIGEN MARKER) (MADDAM) |
| P02774 | GC | VITAMIN D-BINDING PROTEIN (DBP) (VDB) (GC-GLOBULIN) (GROUP-SPECIFIC COMPONENT) |
| Q8NEC7 | GSTCD | GLUTATHIONE S-TRANSFERASE C-TERMINAL DOMAIN-CONTAINING PROTEIN |
| O94988 | FAM13A FAM13A1 KIAA0914 | PROTEIN FAM13A |
| P21781 | FGF7 KGF | FIBROBLAST GROWTH FACTOR 7 (FGF-7) (HEPARIN-BINDING GROWTH FACTOR 7) (HBGF-7) (KERATINOCYTE GROWTH FACTOR) |
| Q9H4Y5 | GSTO2 | GLUTATHIONE S-TRANSFERASE OMEGA-2 (GSTO-2) (EC 2.5.1.18) (GLUTATHIONE S-TRANSFERASE OMEGA 2-2) (GSTO 2-2) (GLUTATHIONE-DEPENDENT DEHYDROASCORBATE REDUCTASE) (EC 1.8.5.1) (MONOMETHYLARSONIC ACID REDUCTASE) (MMA(V) REDUCTASE) (EC 1.20.4.2) |
| P11509 | CYP2A6 CYP2A3 | CYTOCHROME P450 2A6 (EC 1.14.13.-) (1,4-CINEOLE 2-EXO-MONOOXYGENASE) (CYPIIA6) (COUMARIN 7-HYDROXYLASE) (CYTOCHROME P450 IIA3) (CYTOCHROME P450(I)) |
| O94779 | CNTN5 | CONTACTIN-5 (NEURAL RECOGNITION MOLECULE NB-2) (HNB-2) |
| P04141 | CSF2 GMCSF | GRANULOCYTE-MACROPHAGE COLONY-STIMULATING FACTOR (GM-CSF) (COLONY-STIMULATING FACTOR) (CSF) (MOLGRAMOSTIN) (SARGRAMOSTIM) |
| P07099 | EPHX1 EPHX EPOX | EPOXIDE HYDROLASE 1 (EC 3.3.2.9) (EPOXIDE HYDRATASE) (MICROSOMAL EPOXIDE HYDROLASE) |
| P03372 | ESR1 ESR NR3A1 | ESTROGEN RECEPTOR (ER) (ER-ALPHA) (ESTRADIOL RECEPTOR) (NUCLEAR RECEPTOR SUBFAMILY 3 GROUP A MEMBER 1) |
| Q10586 | DBP | D SITE-BINDING PROTEIN (ALBUMIN D BOX-BINDING PROTEIN) (ALBUMIN D-ELEMENT-BINDING PROTEIN) (TAX-RESPONSIVE ENHANCER ELEMENT-BINDING PROTEIN 302) (TAXREB302) |
| P14780 | MMP9 CLG4B | "MATRIX METALLOPROTEINASE-9 (MMP-9) (EC 3.4.24.35) (92 KDA GELATINASE) (92 KDA TYPE IV COLLAGENASE) (GELATINASE B) (GELB) [CLEAVED INTO: 67 KDA MATRIX METALLOPROTEINASE-9; 82 KDA MATRIX METALLOPROTEINASE-9]" |
| P39900 | MMP12 | NA |
| P03956 | MMP1 CLG | "INTERSTITIAL COLLAGENASE (EC 3.4.24.7) (FIBROBLAST COLLAGENASE) (MATRIX METALLOPROTEINASE-1) (MMP-1) [CLEAVED INTO: 22 KDA INTERSTITIAL COLLAGENASE; 27 KDA INTERSTITIAL COLLAGENASE]" |
| A1Z1Q3 | MACROD2 C20ORF133 | O-ACETYL-ADP-RIBOSE DEACETYLASE MACROD2 (EC 3.2.2.-) (EC 3.5.1.-) (MACRO DOMAIN-CONTAINING PROTEIN 2) ([PROTEIN ADP-RIBOSYLGLUTAMATE] HYDROLASE) |

1. Proteins specific HYPERTENSION, ESSENTIAL only

| Uniprot ID | Gene Symbol | Protein Name from UNIPROT |
| --- | --- | --- |
| Q9H4A3 | WNK1 HSN2 KDP KIAA0344 PRKWNK1 | SERINE/THREONINE-PROTEIN KINASE WNK1 (EC 2.7.11.1) (ERYTHROCYTE 65 KDA PROTEIN) (P65) (KINASE DEFICIENT PROTEIN) (PROTEIN KINASE LYSINE-DEFICIENT 1) (PROTEIN KINASE WITH NO LYSINE 1) (HWNK1) |
| Q92574 | TSC1 KIAA0243 TSC | HAMARTIN (TUBEROUS SCLEROSIS 1 PROTEIN) |
| P07101 | TH TYH | TYROSINE 3-MONOOXYGENASE (EC 1.14.16.2) (TYROSINE 3-HYDROXYLASE) (TH) |
| Q7L1I2 | SV2B KIAA0735 | SYNAPTIC VESICLE GLYCOPROTEIN 2B |
| O95619 | YEATS4 GAS41 | YEATS DOMAIN-CONTAINING PROTEIN 4 (GLIOMA-AMPLIFIED SEQUENCE 41) (GAS41) (NUMA-BINDING PROTEIN 1) (NUBI-1) (NUBI1) |
| Q2TBF2 | WSCD2 KIAA0789 | WSC DOMAIN-CONTAINING PROTEIN 2 |
| Q96J92 | WNK4 PRKWNK4 | SERINE/THREONINE-PROTEIN KINASE WNK4 (EC 2.7.11.1) (PROTEIN KINASE LYSINE-DEFICIENT 4) (PROTEIN KINASE WITH NO LYSINE 4) |
| P42898 | MTHFR | METHYLENETETRAHYDROFOLATE REDUCTASE (EC 1.5.1.20) |
| Q96PU5 | NEDD4L KIAA0439 NEDL3 | E3 UBIQUITIN-PROTEIN LIGASE NEDD4-LIKE (EC 6.3.2.-) (NEDD4.2) (NEDD4-2) |
| P08235 | NR3C2 MCR MLR | MINERALOCORTICOID RECEPTOR (MR) (NUCLEAR RECEPTOR SUBFAMILY 3 GROUP C MEMBER 2) |
| P54277 | PMS1 PMSL1 | PMS1 PROTEIN HOMOLOG 1 (DNA MISMATCH REPAIR PROTEIN PMS1) |
| P11086 | PNMT PENT | PHENYLETHANOLAMINE N-METHYLTRANSFERASE (PNMTASE) (EC 2.1.1.28) (NORADRENALINE N-METHYLTRANSFERASE) |
| O43663 | PRC1 | PROTEIN REGULATOR OF CYTOKINESIS 1 |
| Q13976 | PRKG1 PRKG1B PRKGR1A PRKGR1B | CGMP-DEPENDENT PROTEIN KINASE 1 (CGK 1) (CGK1) (EC 2.7.11.12) (CGMP-DEPENDENT PROTEIN KINASE I) (CGKI) |
| P00797 | REN | RENIN (EC 3.4.23.15) (ANGIOTENSINOGENASE) |
| P41220 | RGS2 G0S8 GIG31 | REGULATOR OF G-PROTEIN SIGNALING 2 (RGS2) (CELL GROWTH-INHIBITING GENE 31 PROTEIN) (G0/G1 SWITCH REGULATORY PROTEIN 8) |
| P05091 | ALDH2 ALDM | ALDEHYDE DEHYDROGENASE, MITOCHONDRIAL (EC 1.2.1.3) (ALDH CLASS 2) (ALDH-E2) (ALDHI) |
| P35414 | AGTRL1 | APELIN RECEPTOR (ANGIOTENSIN RECEPTOR-LIKE 1) (G-PROTEIN COUPLED RECEPTOR APJ) (G-PROTEIN COUPLED RECEPTOR HG11) |
| P05026 | ATP1B1 ATP1B | SODIUM/POTASSIUM-TRANSPORTING ATPASE SUBUNIT BETA-1 (SODIUM/POTASSIUM-DEPENDENT ATPASE SUBUNIT BETA-1) |
| P02649 | APOE | APOLIPOPROTEIN E (APO-E) |
| O95180 | CACNA1H | VOLTAGE-DEPENDENT T-TYPE CALCIUM CHANNEL SUBUNIT ALPHA-1H (LOW-VOLTAGE-ACTIVATED CALCIUM CHANNEL ALPHA1 3.2 SUBUNIT) (VOLTAGE-GATED CALCIUM CHANNEL SUBUNIT ALPHA CAV3.2) |
| P30411 | BDKRB2 BKR2 | B2 BRADYKININ RECEPTOR (B2R) (BK-2 RECEPTOR) |
| P01258 | CALCA CALC1 | "CALCITONIN [CLEAVED INTO: CALCITONIN; KATACALCIN (CALCITONIN CARBOXYL-TERMINAL PEPTIDE) (CCP) (PDN-21)]" |
| P35611 | ADD1 ADDA | ALPHA-ADDUCIN (ERYTHROCYTE ADDUCIN SUBUNIT ALPHA) |
| Q9BYF1 | ACE2 UNQ868/PRO1885 | ANGIOTENSIN-CONVERTING ENZYME 2 (EC 3.4.17.23) (ACE-RELATED CARBOXYPEPTIDASE) (ANGIOTENSIN-CONVERTING ENZYME HOMOLOG) (ACEH) (METALLOPROTEASE MPROT15) [CLEAVED INTO: PROCESSED ANGIOTENSIN-CONVERTING ENZYME 2] |
| P35612 | ADD2 ADDB | BETA-ADDUCIN (ERYTHROCYTE ADDUCIN SUBUNIT BETA) |
| P30556 | AGTR1 AGTR1A AGTR1B AT2R1 AT2R1B | TYPE-1 ANGIOTENSIN II RECEPTOR (AT1AR) (AT1BR) (ANGIOTENSIN II TYPE-1 RECEPTOR) (AT1) |
| P01019 | AGT SERPINA8 | "ANGIOTENSINOGEN (SERPIN A8) [CLEAVED INTO: ANGIOTENSIN-1 (ANGIOTENSIN 1-10) (ANGIOTENSIN I) (ANG I); ANGIOTENSIN-2 (ANGIOTENSIN 1-8) (ANGIOTENSIN II) (ANG II); ANGIOTENSIN-3 (ANGIOTENSIN 2-8) (ANGIOTENSIN III) (ANG III) (DES-ASP[1]-ANGIOTENSIN II); ANGIOTENSIN-4 (ANGIOTENSIN 3-8) (ANGIOTENSIN IV) (ANG IV); ANGIOTENSIN 1-9; ANGIOTENSIN 1-7; ANGIOTENSIN 1-5; ANGIOTENSIN 1-4]" |
| P61626 | LYZ LZM | LYSOZYME C (EC 3.2.1.17) (1,4-BETA-N-ACETYLMURAMIDASE C) |
| P20645 | M6PR MPR46 MPRD | CATION-DEPENDENT MANNOSE-6-PHOSPHATE RECEPTOR (CD MAN-6-P RECEPTOR) (CD-MPR) (46 KDA MANNOSE 6-PHOSPHATE RECEPTOR) (MPR 46) |
| O95373 | IPO7 RANBP7 | IMPORTIN-7 (IMP7) (RAN-BINDING PROTEIN 7) (RANBP7) |
| P06870 | KLK1 | KALLIKREIN-1 (EC 3.4.21.35) (KIDNEY/PANCREAS/SALIVARY GLAND KALLIKREIN) (TISSUE KALLIKREIN) |
| P14060 | HSD3B1 3BH HSDB3A | "3 BETA-HYDROXYSTEROID DEHYDROGENASE/DELTA 5-->4-ISOMERASE TYPE 1 (3 BETA-HYDROXYSTEROID DEHYDROGENASE/DELTA 5-->4-ISOMERASE TYPE I) (3-BETA-HSD I) (TROPHOBLAST ANTIGEN FDO161G) [INCLUDES: 3-BETA-HYDROXY-DELTA(5)-STEROID DEHYDROGENASE (EC 1.1.1.145) (3-BETA-HYDROXY-5-ENE STEROID DEHYDROGENASE) (PROGESTERONE REDUCTASE); STEROID DELTA-ISOMERASE (EC 5.3.3.1) (DELTA-5-3-KETOSTEROID ISOMERASE)]" |
| P06213 | INSR | "INSULIN RECEPTOR (IR) (EC 2.7.10.1) (CD ANTIGEN CD220) [CLEAVED INTO: INSULIN RECEPTOR SUBUNIT ALPHA; INSULIN RECEPTOR SUBUNIT BETA]" |
| P80365 | HSD11B2 HSD11K SDR9C3 | CORTICOSTEROID 11-BETA-DEHYDROGENASE ISOZYME 2 (EC 1.1.1.-) (11-BETA-HYDROXYSTEROID DEHYDROGENASE TYPE 2) (11-DH2) (11-BETA-HSD2) (11-BETA-HYDROXYSTEROID DEHYDROGENASE TYPE II) (11-HSD TYPE II) (11-BETA-HSD TYPE II) (NAD-DEPENDENT 11-BETA-HYDROXYSTEROID DEHYDROGENASE) (11-BETA-HSD) (SHORT CHAIN DEHYDROGENASE/REDUCTASE FAMILY 9C MEMBER 3) |
| P32298 | GRK4 GPRK2L GPRK4 | G PROTEIN-COUPLED RECEPTOR KINASE 4 (EC 2.7.11.16) (G PROTEIN-COUPLED RECEPTOR KINASE GRK4) (ITI1) |
| Q8WU20 | FRS2 | FIBROBLAST GROWTH FACTOR RECEPTOR SUBSTRATE 2 (FGFR SUBSTRATE 2) (FGFR-SIGNALING ADAPTOR SNT) (SUC1-ASSOCIATED NEUROTROPHIC FACTOR TARGET 1) (SNT-1) |
| P16520 | GNB3 | GUANINE NUCLEOTIDE-BINDING PROTEIN G(I)/G(S)/G(T) SUBUNIT BETA-3 (TRANSDUCIN BETA CHAIN 3) |
| Q02928 | CYP4A11 CYP4A2 | CYTOCHROME P450 4A11 (20-HYDROXYEICOSATETRAENOIC ACID SYNTHASE) (20-HETE SYNTHASE) (CYP4AII) (CYPIVA11) (CYTOCHROME P-450HK-OMEGA) (CYTOCHROME P450HL-OMEGA) (FATTY ACID OMEGA-HYDROXYLASE) (LAURIC ACID OMEGA-HYDROXYLASE) (EC 1.14.15.3) |
| P20800 | EDN2 | ENDOTHELIN-2 (ET-2) (PREPROENDOTHELIN-2) (PPET2) |
| P19099 | CYP11B2 | CYTOCHROME P450 11B2, MITOCHONDRIAL (ALDOSTERONE SYNTHASE) (ALDOS) (EC 1.14.15.4) (EC 1.14.15.5) (ALDOSTERONE-SYNTHESIZING ENZYME) (CYPXIB2) (CYTOCHROME P-450ALDO) (CYTOCHROME P-450C18) (STEROID 18-HYDROXYLASE) |
| P51589 | CYP2J2 | CYTOCHROME P450 2J2 (EC 1.14.14.1) (ARACHIDONIC ACID EPOXYGENASE) (CYPIIJ2) |
| P32418 | SLC8A1 CNC NCX1 | SODIUM/CALCIUM EXCHANGER 1 (NA(+)/CA(2+)-EXCHANGE PROTEIN 1) (SOLUTE CARRIER FAMILY 8 MEMBER 1) |
| P02730 | SLC4A1 AE1 DI EPB3 | BAND 3 ANION TRANSPORT PROTEIN (ANION EXCHANGE PROTEIN 1) (AE 1) (ANION EXCHANGER 1) (SOLUTE CARRIER FAMILY 4 MEMBER 1) (CD ANTIGEN CD233) |
| Q8NFF2 | SLC24A4 NCKX4 | SODIUM/POTASSIUM/CALCIUM EXCHANGER 4 (NA(+)/K(+)/CA(2+)-EXCHANGE PROTEIN 4) (SOLUTE CARRIER FAMILY 24 MEMBER 4) |
| Q9HC58 | SLC24A3 NCKX3 | SODIUM/POTASSIUM/CALCIUM EXCHANGER 3 (NA(+)/K(+)/CA(2+)-EXCHANGE PROTEIN 3) (SOLUTE CARRIER FAMILY 24 MEMBER 3) |
| P16581 | SELE ELAM1 | E-SELECTIN (CD62 ANTIGEN-LIKE FAMILY MEMBER E) (ENDOTHELIAL LEUKOCYTE ADHESION MOLECULE 1) (ELAM-1) (LEUKOCYTE-ENDOTHELIAL CELL ADHESION MOLECULE 2) (LECAM2) (CD ANTIGEN CD62E) |
| O15539 | RGS5 | REGULATOR OF G-PROTEIN SIGNALING 5 (RGS5) |

1. Proteins specific TUBERCULOSIS, PULMONARY only

| Uniprot ID | Gene Symbol | Protein Name from UNIPROT |
| --- | --- | --- |
| P15260 | IFNGR1 | INTERFERON GAMMA RECEPTOR 1 (IFN-GAMMA RECEPTOR 1) (IFN-GAMMA-R1) (CDW119) (CD ANTIGEN CD119) |
| P42701 | IL12RB1 IL12R IL12RB | INTERLEUKIN-12 RECEPTOR SUBUNIT BETA-1 (IL-12 RECEPTOR SUBUNIT BETA-1) (IL-12R SUBUNIT BETA-1) (IL-12R-BETA-1) (IL-12RB1) (IL-12 RECEPTOR BETA COMPONENT) (CD ANTIGEN CD212) |
| P41968 | MC3R | MELANOCORTIN RECEPTOR 3 (MC3-R) |
| P14174 | MIF GLIF MMIF | MACROPHAGE MIGRATION INHIBITORY FACTOR (MIF) (EC 5.3.2.1) (GLYCOSYLATION-INHIBITING FACTOR) (GIF) (L-DOPACHROME ISOMERASE) (L-DOPACHROME TAUTOMERASE) (EC 5.3.3.12) (PHENYLPYRUVATE TAUTOMERASE) |
| P78504 | JAG1 JAGL1 | PROTEIN JAGGED-1 (JAGGED1) (HJ1) (CD ANTIGEN CD339) |
| Q99572 | P2RX7 | P2X PURINOCEPTOR 7 (P2X7) (ATP RECEPTOR) (P2Z RECEPTOR) (PURINERGIC RECEPTOR) |
| P01210 | PENK | "PROENKEPHALIN-A [CLEAVED INTO: SYNENKEPHALIN; MET-ENKEPHALIN (OPIOID GROWTH FACTOR) (OGF); PENK(114-133); PENK(143-183); MET-ENKEPHALIN-ARG-GLY-LEU; LEU-ENKEPHALIN; PENK(237-258); MET-ENKEPHALIN-ARG-PHE]" |
| P35228 | NOS2 NOS2A | NITRIC OXIDE SYNTHASE, INDUCIBLE (EC 1.14.13.39) (HEPATOCYTE NOS) (HEP-NOS) (INDUCIBLE NO SYNTHASE) (INDUCIBLE NOS) (INOS) (NOS TYPE II) (PEPTIDYL-CYSTEINE S-NITROSYLASE NOS2) |
| P49279 | SLC11A1 LSH NRAMP NRAMP1 | NATURAL RESISTANCE-ASSOCIATED MACROPHAGE PROTEIN 1 (NRAMP 1) (SOLUTE CARRIER FAMILY 11 MEMBER 1) |
| Q92583 | CCL17 SCYA17 TARC | C-C MOTIF CHEMOKINE 17 (CC CHEMOKINE TARC) (SMALL-INDUCIBLE CYTOKINE A17) (THYMUS AND ACTIVATION-REGULATED CHEMOKINE) |
| P17927 | CR1 C3BR | COMPLEMENT RECEPTOR TYPE 1 (C3B/C4B RECEPTOR) (CD ANTIGEN CD35) |
| Q9NNX6 | CD209 CLEC4L | CD209 ANTIGEN (C-TYPE LECTIN DOMAIN FAMILY 4 MEMBER L) (DENDRITIC CELL-SPECIFIC ICAM-3-GRABBING NON-INTEGRIN 1) (DC-SIGN) (DC-SIGN1) (CD ANTIGEN CD209) |
| P13500 | CCL2 MCP1 SCYA2 | C-C MOTIF CHEMOKINE 2 (HC11) (MONOCYTE CHEMOATTRACTANT PROTEIN 1) (MONOCYTE CHEMOTACTIC AND ACTIVATING FACTOR) (MCAF) (MONOCYTE CHEMOTACTIC PROTEIN 1) (MCP-1) (MONOCYTE SECRETORY PROTEIN JE) (SMALL-INDUCIBLE CYTOKINE A2) |
| Q7Z4H7 | HAUS6 DGT6 FAM29A KIAA1574 | HAUS AUGMIN-LIKE COMPLEX SUBUNIT 6 |
| Q9UH73 | EBF1 COE1 EBF | TRANSCRIPTION FACTOR COE1 (O/E-1) (OE-1) (EARLY B-CELL FACTOR) |
| Q8TF09 | DYNLRB2 DNCL2B DNLC2B ROBLD2 | DYNEIN LIGHT CHAIN ROADBLOCK-TYPE 2 (DYNEIN LIGHT CHAIN 2B, CYTOPLASMIC) (ROADBLOCK DOMAIN-CONTAINING PROTEIN 2) |
| Q9UBR2 | CTSZ | CATHEPSIN Z (EC 3.4.18.1) (CATHEPSIN P) (CATHEPSIN X) |
| P19544 | WT1 | WILMS TUMOR PROTEIN (WT33) |
| Q9UIK5 | TMEFF2 HPP1 TENB2 TPEF UNQ178/PRO204 | TOMOREGULIN-2 (TR-2) (HYPERPLASTIC POLYPOSIS PROTEIN 1) (TRANSMEMBRANE PROTEIN WITH EGF-LIKE AND TWO FOLLISTATIN-LIKE DOMAINS) |
| P20333 | TNFRSF1B TNFBR TNFR2 | "TUMOR NECROSIS FACTOR RECEPTOR SUPERFAMILY MEMBER 1B (TUMOR NECROSIS FACTOR RECEPTOR 2) (TNF-R2) (TUMOR NECROSIS FACTOR RECEPTOR TYPE II) (TNF-RII) (TNFR-II) (P75) (P80 TNF-ALPHA RECEPTOR) (CD ANTIGEN CD120B) (ETANERCEPT) [CLEAVED INTO: TUMOR NECROSIS FACTOR RECEPTOR SUPERFAMILY MEMBER 1B, MEMBRANE FORM; TUMOR NECROSIS FACTOR-BINDING PROTEIN 2 (TBP-2) (TBPII)]" |
| Q9BS26 | ERP44 KIAA0573 TXNDC4 UNQ532/PRO1075 | ENDOPLASMIC RETICULUM RESIDENT PROTEIN 44 (ER PROTEIN 44) (ERP44) (THIOREDOXIN DOMAIN-CONTAINING PROTEIN 4) |
| Q03518 | TAP1 ABCB2 PSF1 RING4 Y3 | ANTIGEN PEPTIDE TRANSPORTER 1 (APT1) (ATP-BINDING CASSETTE SUB-FAMILY B MEMBER 2) (PEPTIDE SUPPLY FACTOR 1) (PEPTIDE TRANSPORTER PSF1) (PSF-1) (PEPTIDE TRANSPORTER TAP1) (PEPTIDE TRANSPORTER INVOLVED IN ANTIGEN PROCESSING 1) (REALLY INTERESTING NEW GENE 4 PROTEIN) |
| Q9NR97 | TLR8 UNQ249/PRO286 | TOLL-LIKE RECEPTOR 8 (CD ANTIGEN CD288) |
