# Supplementary material for: Representing and querying disease networks using graph databases
Source: BioData Min. 2016 Jul 25;9:23. doi: 10.1186/s13040-016-0102-8 (PMC4960687; doi:10.1186/s13040-016-0102-8)
Supplement: Additional file 5: — Cypher query on the relationships between a) the O15534 protein (PER gene) and b) the P20393 protein (REV/ERBalpha gene) and the core clock genes, (Table 3). (DOCX 77 kb) [file 13040_2016_102_MOESM5_ESM.docx]

Additional file 5 - Cypher query and figure on the relationships between a) the O15534 protein (PER gene) and b) the P20393 protein (REV/ERBalpha gene) and the core clock genes, (Table 3). The ENTREZ gene names and the protein names are given in Table 5 e and f, respectively (Supplementary file 2).

Cypher query to explore shortest paths (in terms of graph representation) between a) the O15534 protein (PER gene) and b) the P20393 protein (*REV/ERBalpha* gene) and other core clock genes (Table 3). Results are shown in Figure 9.

a) **MATCH** path1=allShortestPaths((p1:Protein{UniprotId: 'O15534'})-[:PPI_ASSOCIATION |SEQ_SIM*]-(p2:Protein))

**WHERE** p2.UniprotId **in** ['O15516', 'O00327','Q16526','Q49AN0','Q99743', 'O15055']

**RETURN** path1

b) **MATCH** path2 = allShortestPaths((p1: Protein{UniprotId : 'P20393'})-[:PPI_ASSOCIATION |SEQ_SIM*..3]-(p2:Protein))

**WHERE** p2.UniprotId **in** ['O15516', 'O00327','Q16526','Q49AN0','Q99743','O15534','O15055']

**RETURN** path2
